# Ribonucleases of Very Stable Multi-Protein Complexes from Different Organs of Sea Cucumber *Paracaudina chilensis*

**DOI:** 10.3390/ijms272110051

**Published:** 2026-11-09

**Authors:** Svetlana E. Soboleva, Irina A. Kostrikina, Pavel S. Dmitrenok, Georgy A. Nevinsky

**Affiliations:** 1 Institute of Chemical Biology and Fundamental Medicine, Siberian Division of Russian Academy of Sciences, pr. Akademika Lavrentieva, 8, Novosibirsk 630090, Russia; soboleva@1bio.ru (S.E.S.); irina@kostrikina.ru (I.A.K.); 2 G. B. Elyakov Pacific Institute of Bioorganic Chemistry, Far Eastern Branch of the Russian Academy of Sciences, 159 Pr. 100 let Vladivostoku, Vladivostok 690022, Russia; paveldmt@piboc.dvo.ru

**Keywords:** sea cucumbers *Paracaudina chilensis*, stable multi-protein complexes, RNase activity of the complexes

## Abstract

Ribonuclease (RNase) activity, which mediates RNA degradation, plays a critical role in the development and survival of various organisms. In this study, we for the first time performed a comprehensive analysis of ribonuclease activity within highly stable multi-protein complexes isolated from five different organs of the holothurian *Paracaudina chilensis* (coelomic fluid, body wall, gut, gonads, and respiratory trees). Previously, these complexes were shown to have molecular masses ranging from 1.2 to 1.8 MDa and to differ in their protein–peptide composition. Using zymography, we identified enzymatically active protein bands with apparent molecular masses ranging from approximately 10 to 180 kDa within these complexes, which exhibited RNase activity at pH values from 3.5 to 9.5. The number of detectable activities differed among organs: the greatest diversity (13 distinct RNA-hydrolyzing activities detected under at least one set of conditions) was observed in the complex from coelomic fluid, whereas the complexes from body wall and gut contained the lowest numbers (5 and 6, respectively). Each complex was characterized by a set of RNase catalytic activities that differed in optimal pH (in the presence of 5 mM MgCl_2_), the number of apparent pH optima per band (from one to four), and sensitivity to metal ions (Mg^2+^, Mn^2+^, Zn^2+^, Co^2+^, Ca^2+^, and Ni^2+^). Some RNase activities were activated by a single metal ion, while others responded to multiple ions. The obtained data demonstrate that preparations of stable multiprotein complexes contain diverse sets of RNA-hydrolyzing activities, but the individual enzymes responsible for these activities remain unidentified. Given the outstanding regenerative capacity of holothurians and the involvement of RNases in the regulation of gene expression and RNA processing, it is suggested that the detected sets of RNase activities may play important roles in tissue regeneration, immune defense, and other physiological processes. This work opens new perspectives for studying the functional role of stable multiprotein complexes in marine invertebrates and highlights the need for further investigation of their catalytic potential.

## 1. Introduction

Several complex mechanisms exist in all living organisms to ensure the detection, elimination, and removal of foreign genetic material, including suppression of its replication and function. Various ribonucleases degrade different types of RNA and also perform a wide range of non-canonical functions, implemented both dependently and independently of their catalytic activity. These functions include a significant role in protein synthesis, epigenetic regulation of gene activity, metabolism of cellular and other RNAs, cell proliferation, regulation of apoptosis, as well as immune defense factors such as antimicrobial and antiviral activity [1,2,3,4,5,6,7]. For eight human RNases, a broad spectrum of biological actions has been described, including neurotoxicity, angiogenesis, anti-pathogenic effects, and immunosuppressive activity [5]. In invertebrates, RNases also exhibit non-canonical activities, particularly participating in innate immune responses [8,9]. For example, in insects, RNases are involved in the degradation of double-stranded RNA as part of antiviral defense, and in the medicinal leech, T2-family RNases have been shown to play a pro-inflammatory role and participate in the regulation of immune responses [10,11].

The current classification of ribonucleases is based on amino acid sequence similarity and enzymatic properties [12]. RNases with catalytic properties and amino acid sequences similar to RNase A [13] are designated as pancreatic-type ribonucleases, whereas enzymes with sequences and catalytic properties resembling bovine kidney RNase K2 [14] are termed non-pancreatic. RNases that are structurally more similar to pancreatic RNases [15,16,17,18,19] but exhibit catalytic properties characteristic of both non-pancreatic and pancreatic RNases [16,17,18,19,20,21,22] have been proposed as mixed-type enzymes. Finally, RNases that remove 3′-terminal mononucleotides from RNA via 2′,3′-cyclic nucleotide intermediates are classified into the RNase A, RNase T1, and RNase T2 families [23]. Microbial RNase T1 has a pH optimum of 7–8, a molecular mass of ~12 kDa, and specificity for guanylic acid. RNase A enzymes, predominantly of vertebrate origin, have a molecular mass of ~14 kDa, a pH optimum of 7–8, and pyrimidine specificity. RNase T2 enzymes, found in animals, plants, and microbes, have molecular masses >20 kDa, pH optima of 4–5, and are generally base-nonspecific [23]. To date, over 100 RNase A homologs have been identified in all tetrapods and fish [24]. More than 20 RNases, including RNase J1, RNase III, and RNase Y, have been identified in *Escherichia coli* and *Bacillus subtilis*, representing Gram-negative and Gram-positive bacteria, respectively; however, only a few of these RNases are common to both organisms [25]. Numerous RNases have been reviewed in [24,25,26].

Despite their evident significance, the biochemical properties of RNases from marine organisms, particularly echinoderms, remain poorly studied. To date, the most detailed information has been obtained for representatives of two other classes of this phylum—sea urchins and sea stars. In sea urchins (*Hemicentrotus pulcherrimus* and *Anthocidaris crassispina*), a T2-type RNase with a pH optimum of 5.0 was detected in the gonads, and its activity sharply increases in female gonads during maturation, suggesting its involvement in sex determination [27]. An RNase with a molecular mass of 37 kDa and a pH optimum of 5.3–5.5 was isolated from eggs of the sea urchin *Psammechinus miliaris* [28,29]. In the sea star *Asterias amurensis*, an enzyme, RNase Aa, also belonging to the T2 family, was isolated from ovaries [30]. This protein, consisting of 231 amino acid residues with a molecular mass of 25.9 kDa, has a pH optimum of 5.0 and preferentially releases guanylic acid from RNA. Phylogenetic analysis showed that RNase Aa occupies an evolutionarily intermediate position between RNases of protozoa and oysters. In addition, two RNases—acidic (pH 5.0) and alkaline (pH 7.4)—with different detergent sensitivities have been described in the sea star *Asterias rubens* [31]. For sea cucumbers, such data were virtually absent until recently. Recently, we for the first time characterized the biochemical properties of RNases from the sea cucumber *Eupentacta fraudatrix* [32]. In the homogenate of this organism, five protein forms with RNase activity were identified, with molecular masses ranging from 15 to 77 kDa. The pH optimum for hydrolysis of a specific microRNA from the related sea cucumber *Apostichopus japonicus* was 5.0, whereas for the nonspecific homooligonucleotide (pA)_23_ it was 6.0. In their characteristics, these enzymes are similar to RNases from other echinoderms.

Moreover, marine invertebrates are of interest due to their remarkable regenerative abilities, unlike vertebrates, which possess only limited regeneration of certain organs, such as epidermis, fins, cornea, and liver [33]. In contrast, some echinoderms, especially holothurians, can rapidly regenerate individual damaged organs or even entire bodies [34,35,36,37]. For example, the sea cucumber *E. fraudatrix*, in addition to intestinal regeneration after evisceration, can completely regenerate an entire organism within a month after being cut into two halves [34,35,36,37]. Therefore, analysis of proteins, enzymes, and other compounds, as well as their biological functions, in these rapidly regenerating sea cucumbers is of particular interest. In particular, during regeneration of the digestive tract of *E. fraudatrix*, enhanced transcription of a number of genes whose products are involved in the regulation of cellular events is observed. These include transcription factors of the Sox family, matrix metalloproteinases (MMPs), their endogenous inhibitors (TIMPs), and PIWI proteins characterized by ribonuclease activity [37]. In addition to protein factors, non-coding RNAs of various classes—circular, long non-coding, and microRNA—make a significant contribution to regenerative morphogenesis. These molecules are involved in the regulation of signaling pathways, proliferative responses, and cell migration [38]. In this regard, it can be assumed that ribonucleases, by hydrolyzing various RNA transcripts, perform a dual function: on the one hand, they provide substrates for biosynthetic processes in regenerating tissues, and on the other, they ensure fine-tuning of gene expression in space and time. Given this functional versatility and the outstanding regenerative capacity of sea cucumbers, the study of their various components, including ribonucleases, is of considerable interest.

In addition to RNases, stable high-molecular-weight protein complexes have been demonstrated in echinoderms—sea urchins and holothurians. Recently, multiprotein complexes were isolated and characterized for the first time from whole organisms and from individual organs and tissues (coelomic fluid, respiratory trees, gonads, gut, and body wall) of the sea cucumber *Paracaudina chilensis* [39]. The complexes from different organs differ in molecular mass (1.2–1.8 MDa) and can be effectively dissociated only in 8.0 M urea with the addition of 0.1 M EDTA and DTT, or in 3.0 M MgCl_2_. Each complex contains numerous proteins (≥10 kDa) and various oligopeptides (≤10 kDa) with the following organ-specific peptide counts: gonads (55) ≤ gut (58) ≤ body wall (64) ≤ coelomic fluid (76) ≤ respiratory trees (104) [39]. Similar stable multiprotein complexes have also been identified in different organs of the sea cucumber *E. fraudatrix* [40], in sea urchin eggs [41], as well as in human milk [42] and placenta [43,44]. These complexes contain various peptides (≤10 kDa) and medium- and high-molecular-mass proteins (≥10 kDa) [40,41,42,43,44]. All such complexes also remain stable in solutions containing 1.5–3.0 M NaCl and only slowly dissociate in the presence of 8.0 M urea [40,41,42,43,44]. It is known that such supramolecular structures are the basis of most biological processes, functioning as molecular machines that perform a wide range of tasks [45]. Due to the spatial proximity of subunits, complexes provide high speed, specificity, and efficiency of substrate interactions, which is critical for metabolic pathways [46,47]. The combination of enzymes and regulatory subunits into a single structure allows coordination of sequential reactions, increased efficiency of substrate interactions, and compartmentalization of biochemical processes [47,48,49,50,51,52,53,54,55,56,57,58,59]. Multiprotein complexes regulate transcription and translation, chromatin organization, intracellular signaling, protein degradation, and the functioning of metabolic cascades. Signaling complexes assembled on receptor domains mediate the activation of phosphorylation cascades and control proliferation, differentiation, and apoptosis. Polyfunctional complexes such as proteasomes and ribosomes perform multiple tasks due to the presence of various active centers and regulatory sites. Of particular interest in this context are the recently discovered stable multiprotein complexes that have no known analogues in the literature.

In the present study, we for the first time used preparations of stable protein complexes isolated from individual organs and tissues of the sea cucumber *P. chilensis*, previously obtained and characterized in [39], for the biochemical investigation of RNases in these complexes. The individual enzymes responsible for these activities remain unidentified, and the term ‘RNase’ is used here operationally to denote RNA-hydrolyzing activity detected by in situ zymography. The objectives of the study included determination of the pH optimum and the effect of metal ions on enzyme activity, as well as identification of individual RNase forms and activities in the protein complexes of different organs of the sea cucumber. Unexpectedly, we found that protein complexes from each organ contain different numbers and types of ribonuclease activities.

## 2. Results

### 2.1. Preparation of Organ Homogenates and Protein Complexes

Samples of whole organisms of the sea cucumber *P. chilensis* and their individual organs (coelomic fluid, upper body wall, respiratory trees, gonads, and gut) were previously subjected to homogenization, and stable protein complexes were isolated as described in [39]. Homogenates of selected organs were fractionated by FPLC gel filtration using Sepharose 4B, efficiently separating different molecules with molecular weights (MWs) of 60–20,000 kDa; representative gel filtration profiles are provided in the Supplementary Data (Figure S1) [39].

As shown earlier, the protein complexes from different organs exhibited distinct MWs ranging from 1.2 to 1.8 MDa: whole organism with all organs, 1.8 ± 0.1; gonads, 1.7 ± 0.2; body wall, 1.7 ± 0.2; gut, 1.5 ± 0.1; respiratory trees, 1.4 ± 0.1; and coelomic fluid, 1.2 ± 0.2 [39]. Complete details of the isolation procedure and characterization of the obtained protein complexes and their components using transmission electron microscopy (Supplementary Figure S2), SDS-PAGE, MALDI mass spectrometry, and other methods are described in [39]. The final preparations of stable multiprotein complexes from all selected organs were used for RNase activity assays.

### 2.2. RNases of Various Organs of Sea Cucumber

**Figure 1:**
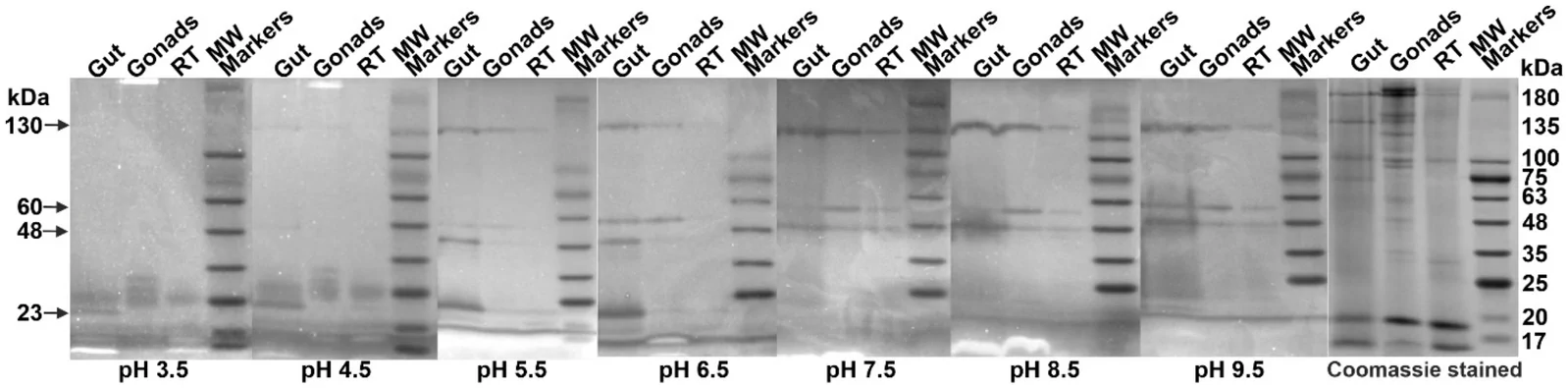
Representative examples of in situ analysis of RNase activity of proteins within stable complexes isolated from the gut, gonads, and respiratory trees (RT) of the sea cucumber. Following SDS-PAGE, gels were incubated in reaction mixtures containing 5.0 mM MgCl_2_ at various pH values. Representative gels (1-pH-unit steps) are shown; the complete pH screen was performed at 0.5-pH-unit increments (pH 3.0–10.0). RNase activity was visualized by ethidium bromide staining, which produced dark bands against a fluorescent background. No activity was detected at pH 3.0 or pH 10.0 The right panels show the corresponding gel fragments stained with Coomassie Brilliant Blue R-250 after development of RNase activity. Molecular masses of marker proteins are indicated on the right (kDa). Additional experimental details are provided in Materials and Methods.

Since several RNases may be present in the complexes, investigation of RNase activity using standard methods (spectrophotometric determination of the enzymatic activity of the complex in solution) would yield a total activity value representing the sum of all enzymes in the complex. Therefore, we chose the in situ activity assay in SDS-PAGE. This method is one of the most effective approaches for detecting ribonucleases that differ in molecular weight (MWs) and catalytic properties within complex biological fluids [60,61,62]. To identify RNases, present in the stable complexes from selected organs, the complexes were incubated with SDS (0.1%) and subjected to SDS-PAGE using gels containing polymerized RNA. After removal of SDS, the RNA-containing gels were incubated in buffers at various pH values (3.5, 4.0, 4.5, 5.0, 5.5, 6.0, 6.5, 7.0, 7.5, 8.0, 8.5, 9.0, 9.5, and 10.0) in the presence of 5.0 mM MgCl_2_. To determine the molecular weights of the RNases, gels were stained with ethidium bromide. Hydrolysis of polymerized RNA occurred in zones corresponding to RNases of different molecular weights and optimal pHs. In gel regions where RNA was degraded into small fragments that were removed during successive washes, dark bands appeared against a uniformly fluorescent background. The relative intensity of each dark band was estimated for each gel lane. For each stable complex, separate SDS-PAGE runs were performed for each pH value in two independent technical experiments. Following assessment of RNA hydrolysis intensity in the two gels corresponding to each pH condition for every complex, pH-activity profiles were generated for each RNase. As an example, Figure 1 presents the in situ SDS-PAGE analysis of RNases from the gut, gonads, and respiratory tree complexes. Equivalent data were obtained for all five complexes from different organs of the sea cucumber across the entire range of pH values tested.

Control experiments were performed using polyacrylamide gels containing copolymerized RNA but no protein complexes, to which various buffers, metal ions, or EDTA were added (Supplementary Figures S5 and S6). No RNA hydrolysis was detected in any of these gels after SDS-PAGE.

Following in situ SDS-PAGE, the molecular masses of protein bands associated with RNA hydrolysis were compared, and their relative RNase activities were evaluated.

### 2.3. pH Dependencies of RNases of Stable Protein Complexes from Different Organs

Ribonucleases from various organs of diverse organisms catalyze RNA hydrolysis over a broad range of optimal pH values, extending from 3 to 10 [10,11,12,13,14,15,16,17,18,19,20,21,22,23,24,25,26,27,28,29,30]. The efficiency of polymeric RNA hydrolysis was analyzed using the in situ SDS-PAGE method for stable complexes isolated from five organs of the sea cucumber *Paracaudina chilensis*. Each complex was subjected to SDS-PAGE, and the corresponding gels for each organ were incubated in reaction mixtures at different pH values ranging from 3 to 10. The relative activity of RNases with different molecular weights was estimated from the intensity of dark bands.

**Figure 2:**
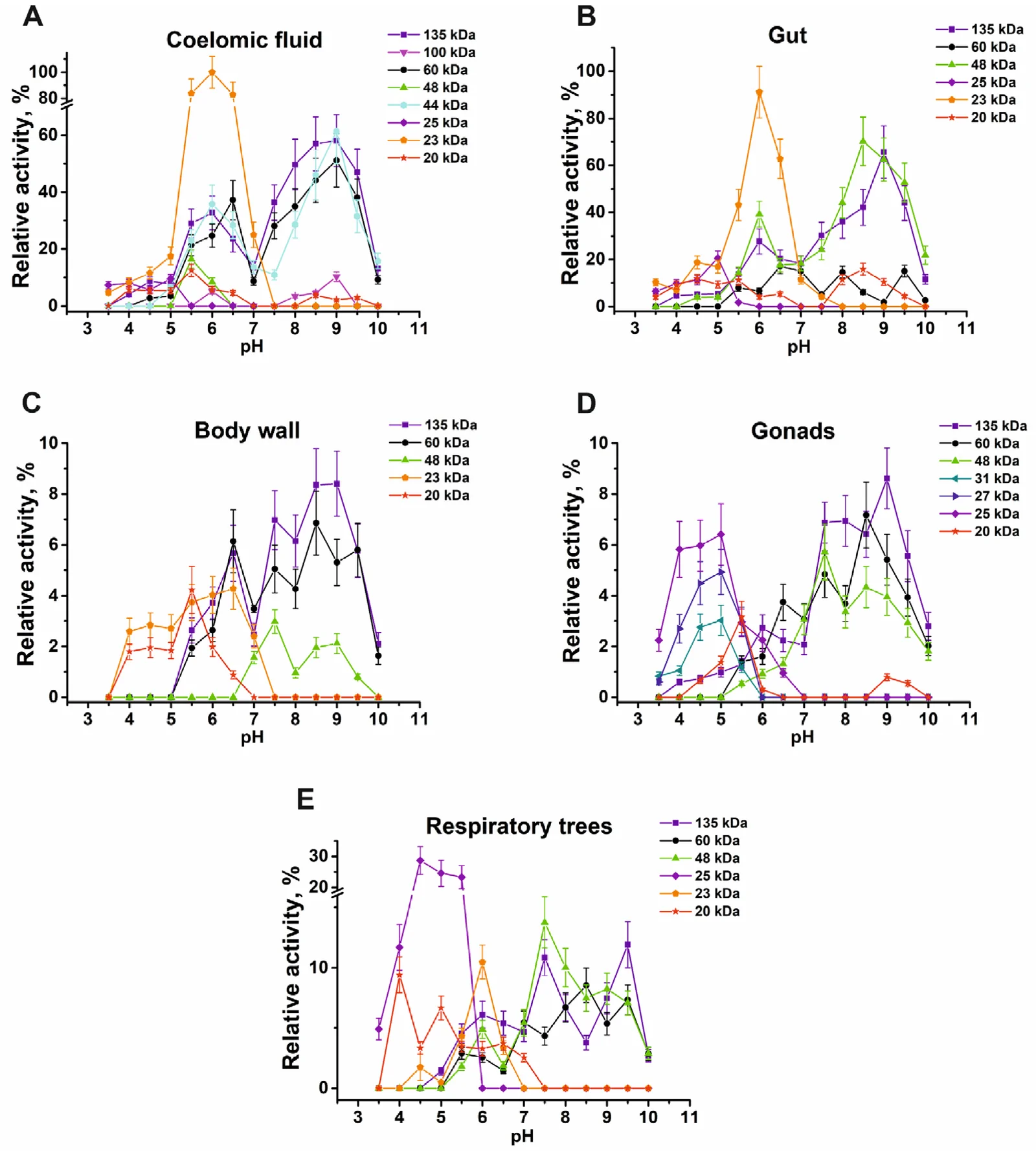
Average relative RNase activities of protein complexes from coelomic fluid (**A**), gut (**B**), body wall (**C**), gonads (**D**), and respiratory trees (**E**), showing enzymes of different molecular weights in RNA hydrolysis at various pH values, as determined by in situ SDS-PAGE analysis in the presence of 5.0 mM MgCl_2_. Data represent averages of two technical replicates for each complex and each pH value. Relative activities are expressed as percentages relative to the activity of the RNase from the coelomic fluid with a molecular weight of ~23 kDa, which was set at 100%. Results are shown as mean ± SD from two experiments. All profiles differed significantly in amplitude, position of maxima, width of the active pH range, and overall shape of the curves (*p* < 0.0001, two-way ANOVA).

Initial attempts to determine optimal pH values for RNA hydrolysis in the absence of metal ions proved challenging. Therefore, MgCl_2_ at a concentration of 5.0 mM was added to the reaction mixtures during the initial screening for optimal pH conditions. The optimal pH values of metal-independent RNases within the complexes from various organs were assessed in a subsequent phase of the study (see below). Averaged data for each pH value and for each enzyme within the complex that hydrolyzed RNA in the presence of 5.0 mM MgCl_2_, grouped by molecular weight, are presented in Figure 2. Samples were treated with 0.1% SDS at 23 °C without heating or reducing agents. Under these conditions, the stable multiprotein complexes may not be fully dissociated. Therefore, some detected bands may represent subcomplexes or co-migrating proteins, and all molecular masses mentioned later in the work reported here are apparent. To convert the raw densitometry values, the intensity of the 23 kDa RNase spot from the coelomic fluid at pH 6.0 was taken as 100% activity, being the maximum among the enzymes from all organs. Statistical analysis is described in Section 4.4; significant differences are indicated on the panels.

The highest number of distinct protein bands exhibiting RNase activity—eight—was detected in the complex from coelomic fluid (Figure 2A). Slightly fewer, seven, were found in gonads, six in both gut and respiratory trees, and the lowest number of RNase activities—five—was observed in the body wall complex (Figure 2B–E). When the bands detected in the metal-dependence series (Section 2.4) are taken into account, the total numbers of distinct apparent-MW bands per organ are 13 in coelomic fluid, 8 in gonads, 6 each in gut and respiratory trees, and 5 in body wall; all bands and the conditions of their detection are compiled in the unified inventory in Table 1. In the complex of each organ, RNase activities with pH optima in the alkaline range (8.0–9.0) were present, whereas the positions of the optima in the acidic and neutral regions differed between complexes from different organs. Overall, RNases from coelomic fluid and gut appeared to be more active than those from body wall and gonads, with relative activities of 60–100% and 6–8%, respectively (Figure 2A–D). The enzymatic activity of protein bands from the respiratory tree complex was intermediate, ranging from 10 to 30% (Figure 2E). In the complexes of all five organs, four active protein bands with identical molecular weight were detected: approximately 135, 60, 48, and 20 kDa. Bands of ~25 and ~23 kDa with RNase activity were present in four organs: the ~25 kDa RNase was absent in the body wall, while the ~23 kDa band was not found in gonads. Four additional RNases were found exclusively in the complex of a single organ: protein bands of ~100 and ~44 kDa in coelomic fluid, and ~31 and ~27 kDa in gonads.

**Figure 3:**
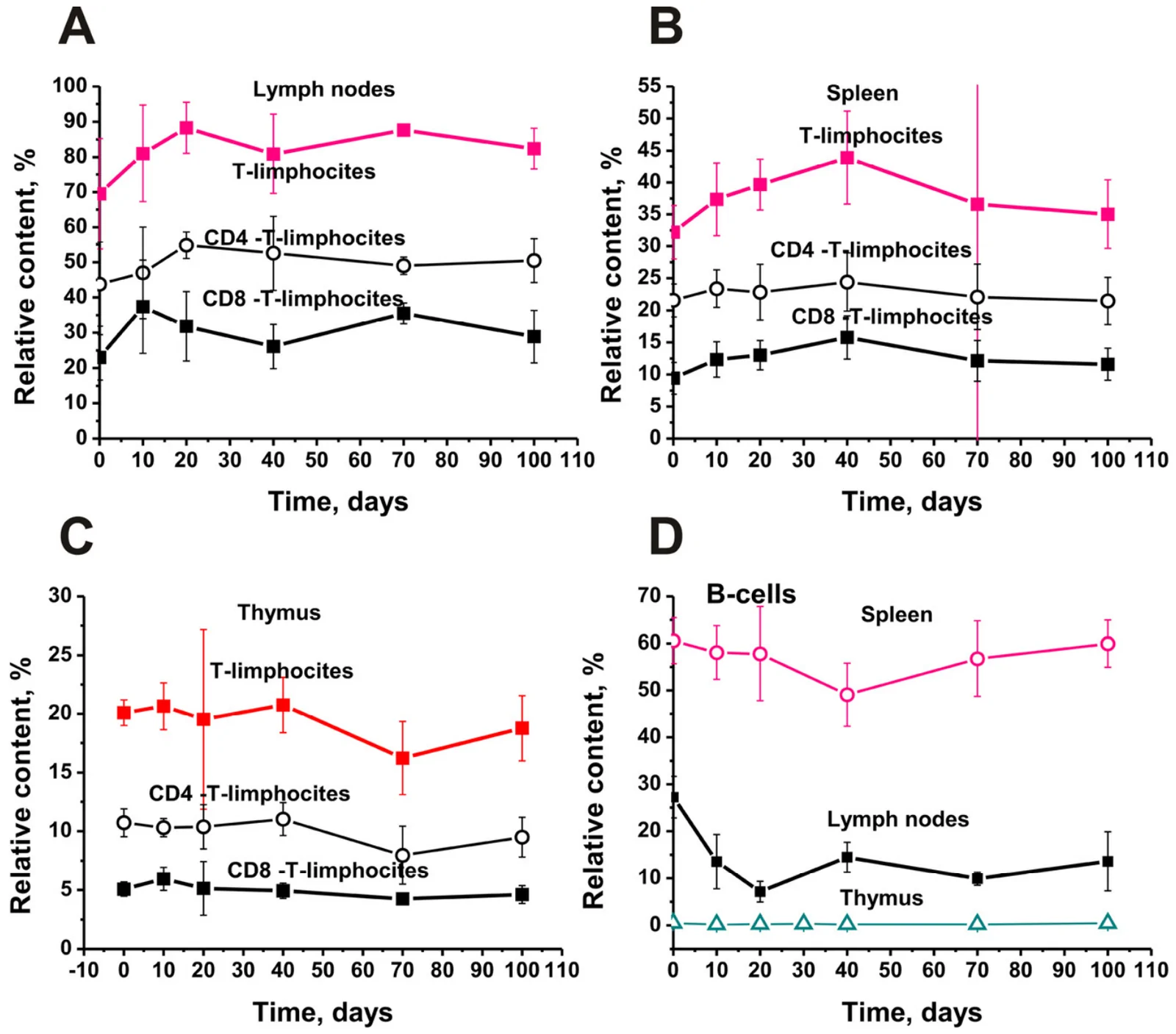
Average data on relative activity of RNases with different MWs having different optimal pHs in the complexes from various organs: 135 kDa (**A**), 60 kDa (**B**), 48 kDa (**C**), 25 kDa (**D**) of protein complexes from coelomic fluid (Figure 2A), gut (Figure 2B), body wall (Figure 2C), gonads (Figure 2D), and respiratory trees (Figure 2E), showing enzymes of different molecular weights in RNA hydrolysis at various pH values, as determined by in situ SDS-PAGE analysis in the presence of 5.0 mM MgCl_2_. Data represent averages of two technical replicates for each complex and each pH value. Relative activities are expressed as percentages relative to the activity of the RNase from the coelomic fluid with a molecular weight of ~23 kDa, which was set at 100%. Results are shown as mean ± SD from two experiments. All profiles differed significantly in amplitude, position of maxima, width of the active pH range, and overall shape of the curves (*p* < 0.0001, two-way ANOVA).

Figure 3 summarizes the RNase activities identified in complexes from the different organs of the sea cucumber.

All five complexes contained an RNase with an MW of ~135 kDa, with relatively low activity (~8–10%) observed only in the body wall, gonads, and respiratory tree complexes. For the ~135 kDa protein band in complexes from coelomic fluid and gut, two pH optima (approximately 6.0 and 9.0) were observed, along with the highest activity (~60%). In contrast, the ~135 kDa band from body wall, gonads, and respiratory trees exhibited three optima at pH 7.5, 8.0, and 9.0 (Figure 3A). The ~60 kDa protein band displayed high activity (~15–50%) with several pH optima (6.5 and 8.0–9.0) in complexes from coelomic fluid and gut (Figure 3B). The activity of this RNase in complexes from body wall, gonads, and respiratory trees was relatively low (~6–9%), though the pH optima were similar. In the gut complex, the ~48 kDa protein band demonstrated high activity (70%) with two pH optima at approximately 6.0 and 8.5, whereas in gonads, body wall, and respiratory trees, the optima were at 7.5 and 9.0 (Figure 3C). Interestingly, in the coelomic fluid complex, the ~48 kDa band exhibited activity only at pH 5.5.. This same RNase, but with lower activity, was also detected in the respiratory tree complex. The RNase was not detected in the gonad complex (Figure 2D). The ~20 kDa protein band showed moderate activity (~4–16%) in the coelomic fluid (pH 5.5 and 8.5), respiratory trees (pH 4.0), and gut (pH 4.5–5.5 and 8.5) complexes. Highly active RNases with a MW of ~44 kDa were found exclusively in the coelomic fluid complex, with optimal pH values of 6.0 and 9.0 (Figure 2A). The ~25 kDa protein bands were detected in all complexes except the body wall; these enzymes exhibited moderate activity (~6–30%) at pH optima in the range of 4.5–5.5 (Figure 3D). Only in the coelomic fluid complex was weak RNase activity observed in a ~100 kDa protein band, with optimal pH values of 6.0 and 9.0 (Figure 2A). The gonad complex uniquely contained two minor RNases of ~31 and 27 kDa, which hydrolyzed RNA with approximately equal activity at pH 4.5–5.0 (Figure 2D). It should be noted that all pH profiles were obtained in the presence of 5.0 mM MgCl_2_. Therefore, the pH optima reported here are apparent and may be influenced by Mg^2+^ binding, especially for activities that are inhibited by Mg^2+^. To compare the optimal pH values (in the presence of 5 mM MgCl_2_) and relative RNase activities in complexes from different organs, all the data described above are summarized in Table 1.

Protein bands with molecular weights of approximately 100 and 44 kDa, exhibiting RNase activity, were detected in this series of experiments only in the complex from coelomic fluid, while the ~27 and ~31 kDa bands were found only in the gonads. Some RNases corresponding to the same protein band were identified in protein complexes from multiple organs. However, in only a few cases did bands of the same molecular weight display a single optimal pH value. In most instances, two to four apparent optimal pH values corresponded to the same protein band (Table 1); some of these may represent a single broad optimum sampled at adjacent pH points or buffer-specific effects, particularly where the optima fall within different buffer systems. For example, the ~20 kDa protein band from complexes of coelomic fluid, body wall, and gonads exhibited two optimal pH values, whereas that from respiratory trees and gut exhibited four. Moreover, these optimal pH values for the ~20 kDa band differed among complexes from various organs. No complete concordance of optimal pH values was observed for protein bands of other molecular weights either. Furthermore, even when optimal pH values for bands of the same molecular weight from different organ complexes coincided, marked differences in their relative RNase activities were evident. For instance, in the gut and coelomic fluid complexes, the ~23 kDa nuclease was substantially more active (91–100%) compared to that from complexes of other organs (2.7–10.0%). RNases within each protein complex from different organs exhibited significant differences in the relative activity of protein bands with identical molecular weights. This finding suggests that complexes from distinct organs may contain RNase activities of similar molecular weights but with different enzymatic properties. Additionally, it is possible that some of these may represent the same enzyme, differing only in the extent of glycosylation, phosphorylation, or other post-translational modifications. Nevertheless, the data described above indicate that in each organ, the stable complex preparation (1.2–1.8 MDa) displays a distinct set of RNA-hydrolyzing activities.

As shown in Table 1, numerous optimal pH values were observed for the different nucleases within stable complexes from various organs. The most frequently occurring optimal pH values fell within the ranges of 5.0–6.0, particularly 5.5, as well as 8.0–9.0.

### 2.4. Dependencies of RNases from Stable Complexes of Coelomic Fluid on Metal Ions

**Figure 4:**
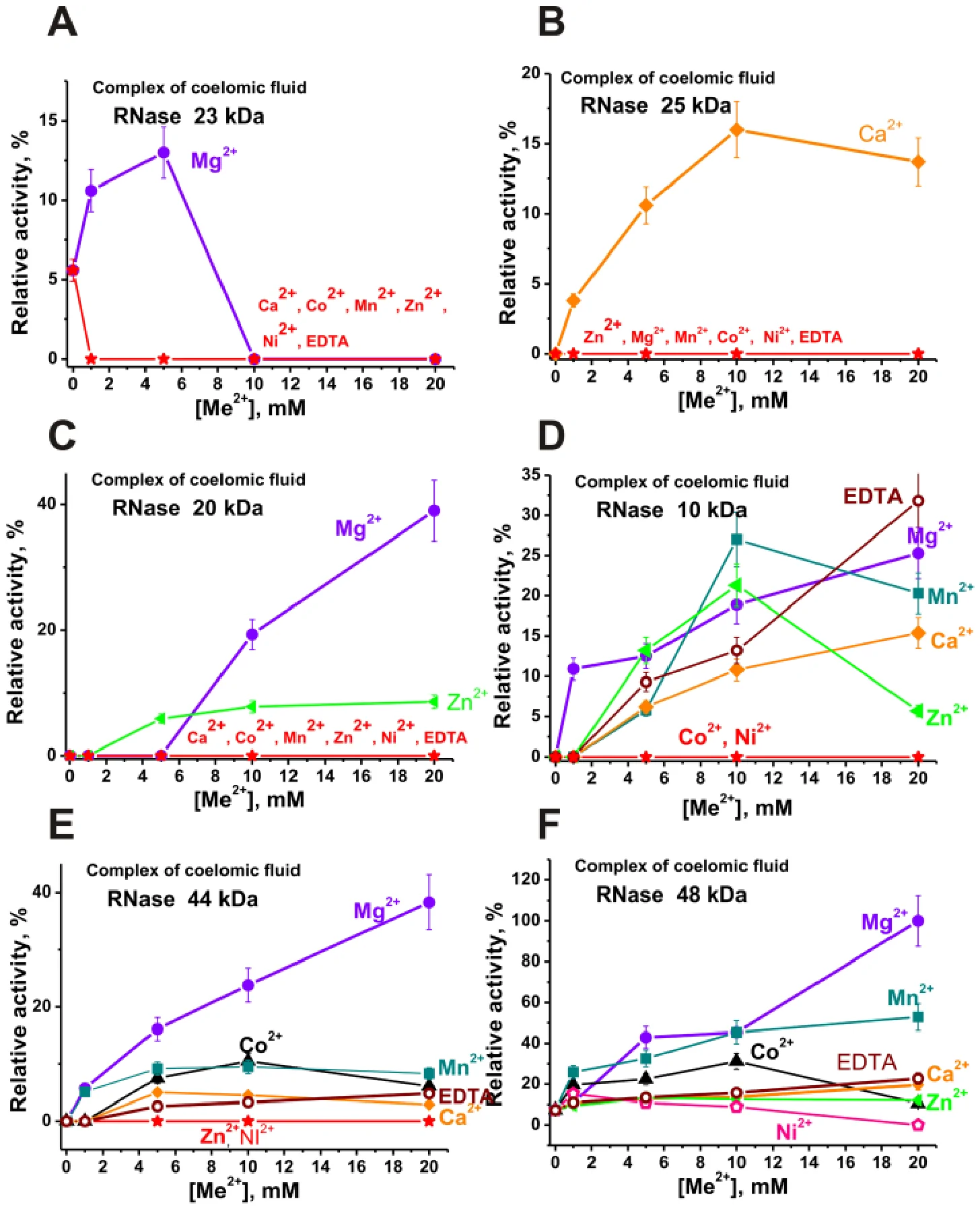
Average data showing the dependence of relative RNase activity at pH 5.5 on the concentration of various metal ions and EDTA for enzymes of different molecular weights present within the protein complex of the coelomic fluid (kDa): 23 (**A**), 25 (**B**), 20 (**C**), 10 (**D**), 44 (**E**), and 48 (**F**). The data were obtained using in situ SDS-PAGE analysis (two gels per pH and per concentration of each metal ion and EDTA tested). In cases where no dependence of RNase activity on one or more of the tested metal ions was observed, all such data are presented together as a single red curve marked with asterisks. The intensity of the 48 kDa RNase spot at 20 mM MgCl_2_ was taken as 100% activity (Panel (**F**)), as it represented the highest value among the enzymes in this experiment. Results are shown as mean ± SD from two experiments. All profiles differed significantly in positions of the maxima, amplitude, and shape of the curves (*p* < 0.0001, two-way ANOVA).

Metal-dependent RNases typically exhibit increased activity in the presence of Mg^2+^ and, in some cases, other divalent cations at concentrations ranging from 1.0 to 10.0 mM [1,2,3,4,5,6,7,8,9,10,11,12,13,14,15,16,17,18,19,20,21,22,23,24,25,26,27,28,29,30,31,32]. A comprehensive assessment of RNase activity within stable complexes from various organs, examining the effects of different metal ions across all optimal pH values, was not feasible. Maximum RNase activity is most frequently observed at pH 5.5, a condition under which nucleases with optima in the pH range of 5 to 6 display substantial activity (Figure 2). A second zone of high RNase activity corresponds to the pH range of 8 to 9. Figure 4 presents the dependence of RNase activity of bands having different molecular weights from coelomic fluid complexes on metal ion concentration and EDTA at pH 5.5. In this section, the metal ion dependence of RNase activities was analyzed using a different set of conditions than in Section 2.3. Instead of a fixed 5.0 mM MgCl_2_, various metal ions (Mg^2+^, Mn^2+^, Zn^2+^, Co^2+^, Ca^2+^, Ni^2+^) were tested across a concentration range of 0–20 mM, along with EDTA. This change in conditions affected the apparent number of active bands, as some RNases that were weakly active with Mg^2+^ alone became detectable with other metal ions, while others lost activity. The band counts in this section therefore differ from those in Section 2.3.

It should be considered that multiple RNases with similar molecular weights may comigrate within a single protein band on the gel. In such cases, the RNases corresponding to that band may be activated by different metal ions and exhibit distinct optimal concentration ranges. As shown in Figure 4A, the ~23 kDa protein band corresponds to a single Mg^2+^-dependent RNase (optimal [MgCl_2_] = 1–5 mM); other metal ions failed to activate this enzyme. The ~25 kDa protein (Figure 4B) was activated only by calcium ions (optimal CaCl_2_ = 10 mM). The ~20 kDa protein band (Figure 4C) showed stronger activation by Mg^2+^ (10–20 mM) than by Zn^2+^ (5.0 mM). The ~10 kDa protein band most likely contains multiple RNases activated by different metal ions, with the exception of Co^2+^ and Ni^2+^ (Figure 4D): Mg^2+^ (1–20 mM), Mn^2+^ (10 mM), Zn^2+^ (10 mM), and Ca^2+^ (20 mM). Notably, the activity of the ~10 kDa RNase(s) increased with increasing EDTA concentration. The increase in activity observed with EDTA may reflect chelation of divalent cations, including trace metal ions present in the assay system and/or metal ions associated with the complexes. However, the present data do not allow us to distinguish between these possibilities.

As previously demonstrated for a stable complex from the sea cucumber *Eupentacta fraudatrix*, such complexes contain various metal ions associated with proteins and other components [63]. Accordingly, it is reasonable to assume that complexes from different organs of *P. chilensis* also can contain metal ions bound to various complex components, including ribonucleases. The addition of EDTA may remove metal ions from the complex and from metal-independent RNases, thereby leading to increased activity.

A similar pattern was observed for several RNases of other molecular weights (see below). The ~44 kDa protein band (Figure 4E) was activated by all metal ions tested except Zn^2+^ and Ni^2+^, with different optimal salt concentrations: Mg^2+^ (1–20 mM), Mn^2+^ (5–10 mM), Co^2+^ (10 mM), and Ca^2+^ (5 mM). The ~44 kDa RNase(s) were also activated by EDTA, though to a lesser extent than the ~10 kDa enzyme(s). The ~48 kDa RNase(s) were reliably activated by all metal ions tested, with the following relative effectiveness: Mg^2+^ (5–20 mM) > Mn^2+^ (1 mM) > Co^2+^ (1 mM) > Ca^2+^ (1 mM) ≈ Zn^2+^ (1 mM) ≈ Ni^2+^ (1–2 mM).

**Figure 5:**
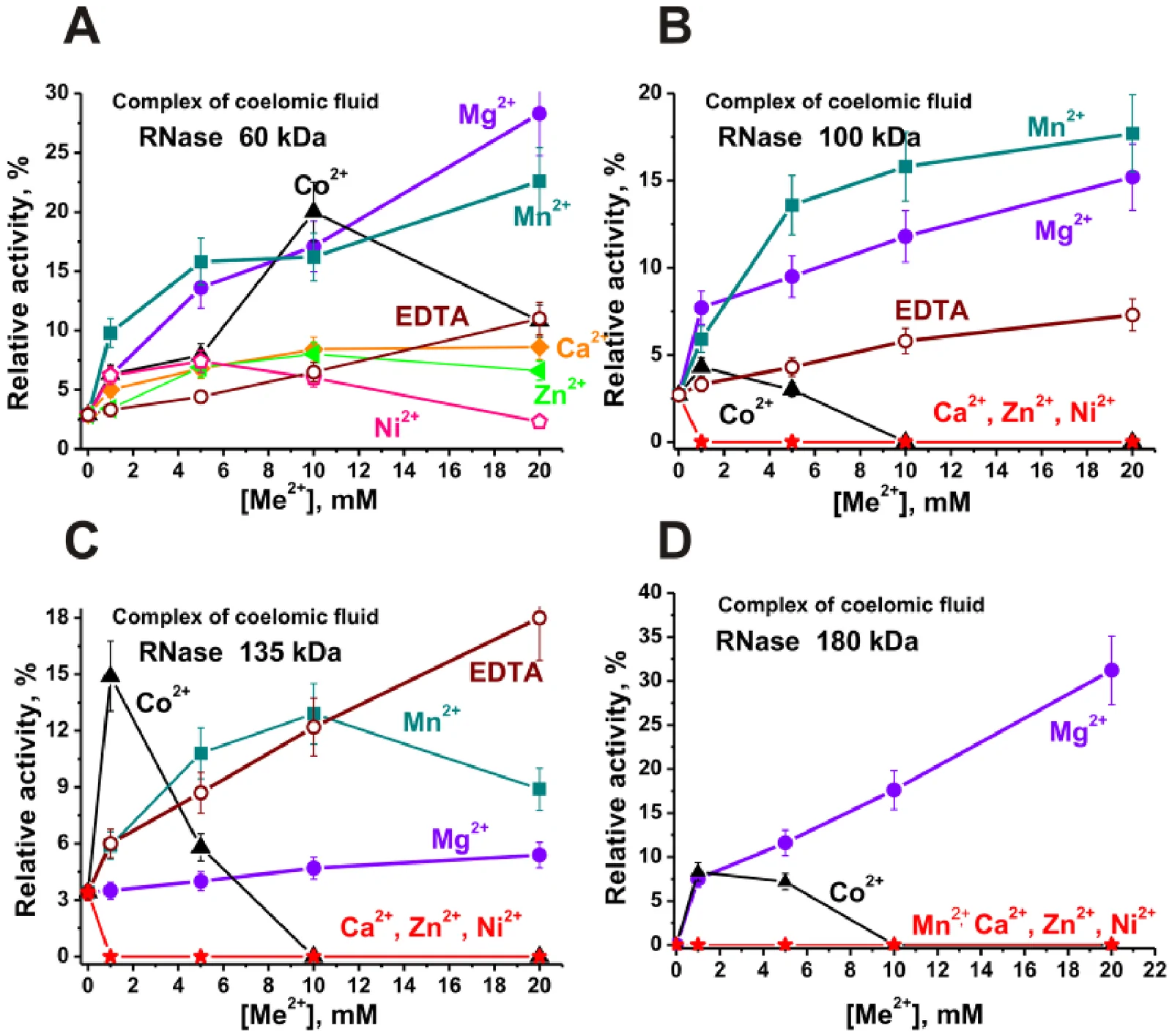
Average data showing the dependence of relative RNase activity at pH 5.5 on the concentration of various metal ions and EDTA for enzymes with molecular weights greater than 60 kDa present within the protein complex of the coelomic fluid (kDa): 60 (**A**), 100 (**B**), 135 (**C**), and 180 (**D**). The data were obtained using in situ SDS-PAGE analysis (two gels for each concentration of every metal ion and EDTA tested). In cases where no RNase activity was observed in response to one or more of the tested metal ions, all such data are presented together as a single red curve marked with asterisks. The intensity of the 48 kDa RNase spot at 20 mM MgCl_2_ was taken as 100% activity (Figure 4F), as it represented the highest value among the enzymes in this experiment. Results are shown as mean ± SD from two experiments. All profiles differed significantly in positions of the maxima, amplitude, and shape of the curves (*p* < 0.0001, two-way ANOVA).

Figure 5 shows the dependence of RNase activity of the coelomic fluid complex on the concentration of various metal ions and EDTA at pH 5.5 for enzymes with molecular weights greater than 60 kDa.

The ~60 kDa RNase(s) (Figure 5A) were activated by various metal ions, with the following relative effectiveness: Mg^2+^ (5–20 mM) ≈ Mn^2+^ (5 mM) ≈ Co^2+^ (10 mM) > Ca^2+^ (10 mM) ≈ Zn^2+^ (10 mM) ≈ Ni^2+^ (5 mM). Weak activation by EDTA was also observed. The ~100 kDa enzyme(s) (Figure 5B) demonstrated activation by Mn^2+^ (5–20 mM) ≈ Mg^2+^ (1–20 mM) > Co^2+^ (1 mM); however, their activity was suppressed by Ca^2+^, Zn^2+^, and Ni^2+^ ions, while the addition of EDTA resulted in increased activity. One possible explanation is that EDTA chelates divalent cations that partially suppress the activity of these enzymes. However, because EDTA can also chelate trace metals from the gel and buffers, this interpretation remains tentative. The strongest increase in activity of the ~135 kDa RNase(s) (Figure 5C) occurred in the presence of Co^2+^ ions (1 mM), followed by Mn^2+^ (10 mM) > Mg^2+^ (1–20 mM). Strong activation by EDTA and suppression of activity by Ca^2+^, Zn^2+^, and Ni^2+^ ions were also observed. The ~180 kDa RNase (Figure 5D) did not exhibit appreciable activity in the absence of metal ions and was activated only by Mg^2+^ (1–20 mM) and Co^2+^ (1 mM).

**Figure 6:**
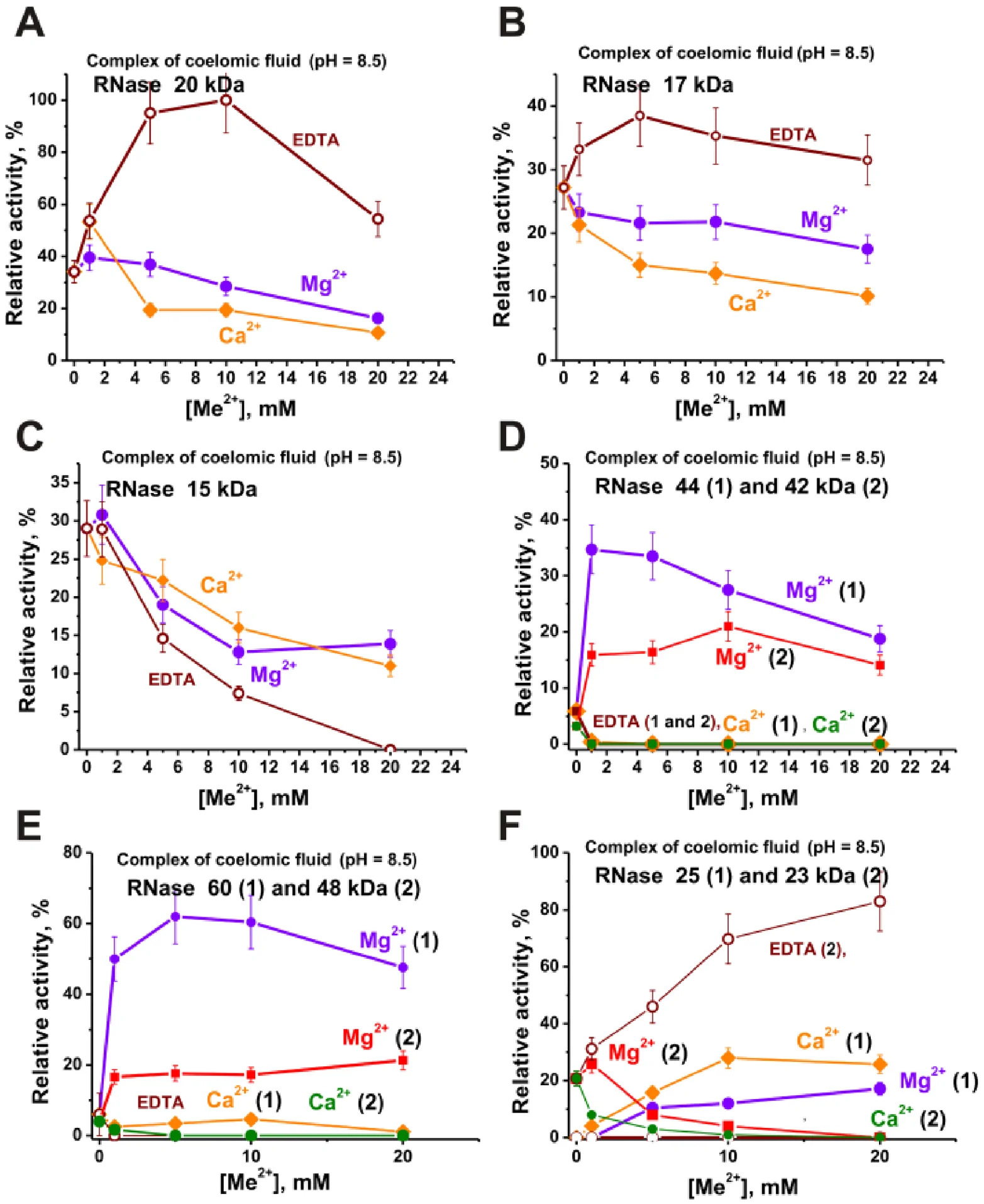
Average data showing the dependence of relative RNase activity at pH 8.5 on the concentration of Mg^2+^ and Ca^2+^ ions, as well as EDTA, for enzymes of different molecular weights present within the protein complex of the coelomic fluid (kDa): 20 (**A**), 17 (**B**), 15 (**C**), 42 and 44 (**D**), 48 and 60 (**E**), and 23 and 25 (**F**). The data were obtained using in situ SDS-PAGE analysis (two gels for each concentration of every metal ion and EDTA tested). The intensity of the 20 kDa RNase spot at 10 mM EDTA was taken as 100% activity (Panel **A**), as it represented the highest value among the enzymes in this experiment. Results are shown as mean ± SD from two experiments. All profiles differed significantly in positions of the maxima, amplitude, and shape of the curves (*p* < 0.0001, two-way ANOVA).

The second important zone of optimal pH values was observed in the range of 8–9 (Figure 2). Accordingly, a pH of 8.5 was selected for analyzing the optimal metal ion concentrations, as this pH supports high or significant activity of nucleases with optimal pH values of 8.0, 8.5, and 9.0. Mg^2+^ and Ca^2+^ ions were used as potentially important coenzymes for these nucleases. Upon analyzing the dependence of RNase activity on the concentration of these ions, nine protein bands exhibiting activity at pH 8.5 were identified. Figure 6 presents the dependence of RNase activity for these nine protein bands on the concentration of Mg^2+^ and Ca^2+^ ions, as well as EDTA.

Interestingly, at pH 8.5, four of the nine protein bands contained metal-independent RNases. Specifically, nucleases with molecular weights of ~20 kDa (Figure 6A) and ~17 kDa (Figure 6B) exhibited high activity. Both nucleases were inhibited by Mg^2+^ and Ca^2+^ ions. It should be assumed that these nucleases are associated with certain metal ions that suppress their activity, and the addition of EDTA relieves this suppression, leading to increased enzymatic activity. The data for the ~15 kDa protein band (Figure 6C) were somewhat unexpected. On one hand, similar to the ~20 kDa and ~17 kDa nucleases, RNA hydrolysis by the ~15 kDa RNase was suppressed by Mg^2+^ and Ca^2+^ ions. At the same time, however, EDTA also suppressed the activity of this ~15 kDa band (Figure 6C). It is possible that the activity observed for this protein band represents the combined activity of both metal-independent and metal-dependent RNases. In such a scenario, if the cofactor for the metal-dependent RNase is, for example, zinc, manganese, cobalt, or nickel, its activity could be suppressed by both Mg^2+^ and Ca^2+^ ions as well as by EDTA.

The protein bands at ~42 kDa and ~44 kDa exhibited weak activity in the absence of metal ions; both enzymes were strongly activated by Mg^2+^ ions, while Ca^2+^ and EDTA inhibited their activity (Figure 6D). It should be noted that despite the small difference in their molecular weights, these protein bands were clearly resolved on the gel. Nevertheless, it remains possible that both proteins represent the same nuclease differing only in the type of glycosylation or other post-translational modifications. The protein bands at ~48 kDa and ~60 kDa corresponded to RNases activated by Mg^2+^ ions; Ca^2+^ weakly activated the ~60 kDa enzyme but inhibited the activity of the ~48 kDa RNase (Figure 6E). EDTA had only a modest effect on the activity of these enzymes. Figure 6F presents data for the metal-independent RNase at ~23 kDa and the enzyme at ~25 kDa, which exhibited no activity in the absence of metal ions. The activity of the ~23 kDa enzyme was inhibited by Mg^2+^ and Ca^2+^ ions but activated by EDTA. In contrast, the ~25 kDa enzyme was activated more effectively by Ca^2+^ than by Mg^2+^, and EDTA did not affect its activity.

It is important to note that two additional protein bands at pH 5.5 (~10 and ~180 kDa) and three additional protein bands at pH 8.5 (~42, ~17 and ~15 kDa) were detected in the coelomic fluid complex under these metal-dependence conditions (Figure 4D, 5D and 6B–D), whereas they were not reported in Section 2.3. This is because the pH-dependence and metal-dependence series were performed on independently prepared gels and normalized to different reference bands; at low relative activities (approximately 5–10%), the detection of these minor bands was not reproducible between independently prepared gel batches. Relative band intensities, and the sets of minor bands detected near the sensitivity limit, are therefore not quantitatively comparable between the two experimental series.

As shown in Figure 2 and Figure 3, RNases present in complexes from different organs can exhibit widely varying optimal pH values, ranging from 3.5 to 9.5. Analysis of the dependence of activity on pH, as well as on the type and concentration of various metal ions, using in situ SDS-PAGE is highly informative but also extremely labor-intensive. Given this, it was not feasible to conduct a complete analysis of RNase activities at all observed optimal pH values (from 3.5 to 9.5) and for all possible metal ions. Therefore, the analysis of RNase dependencies within stable protein complexes from other organs of the sea cucumber was carried out only at pH 5.5 or 6.0.

### 2.5. RNase Activity of Protein Complexes from Body Wall and Gut

**Figure 7:**
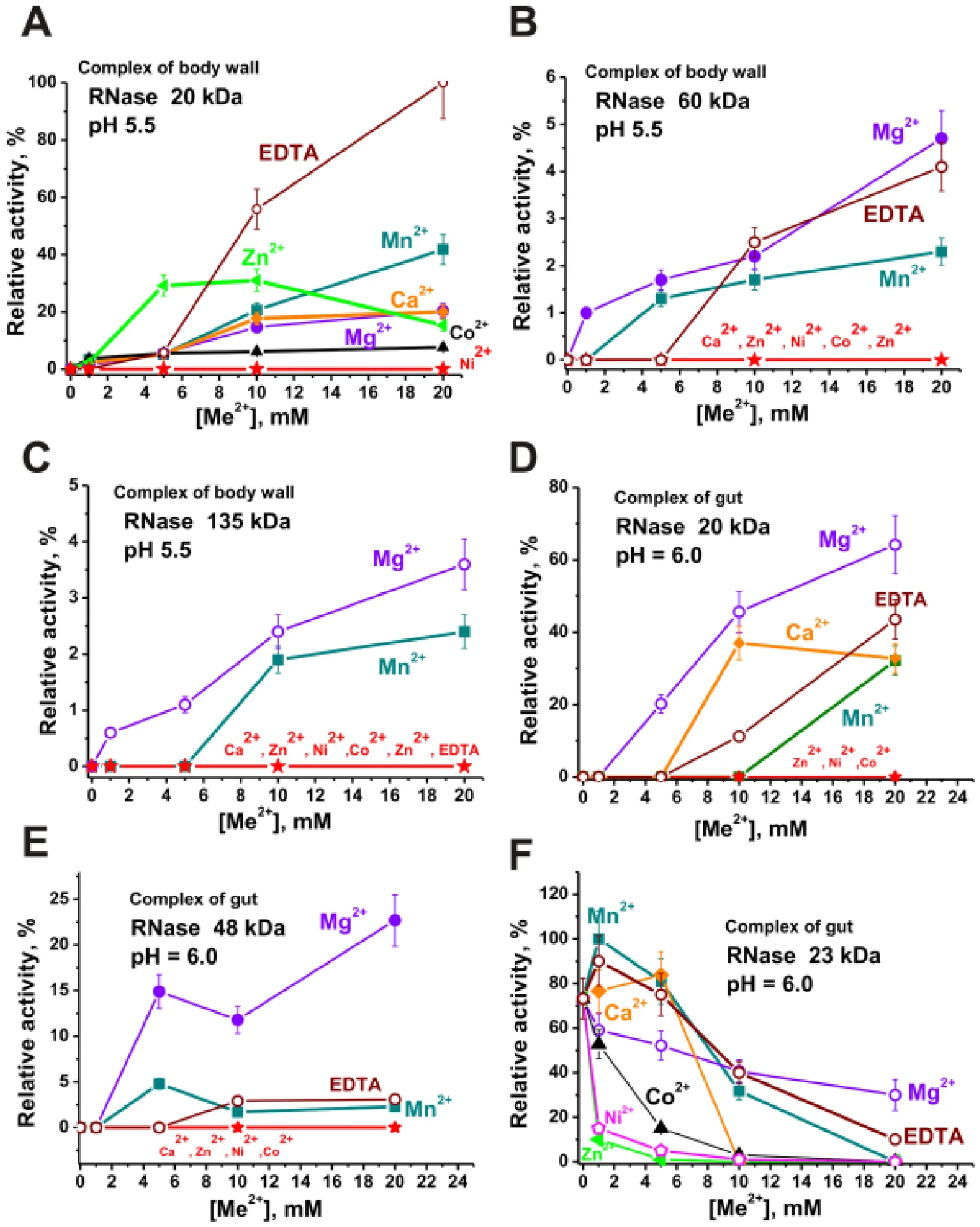
Average data showing the dependence of relative RNase activity on the concentration of various metal ions and EDTA for enzymes of different molecular weights present within the protein complex of the body wall at pH 5.5 (kDa): 20 (**A**), 60 (**B**), and 135 (**C**); and of the gut at pH 6.0 (kDa): 20 (**D**), 48 (**E**), and 23 (**F**). The data were obtained using in situ SDS-PAGE analysis (two gels for each concentration of every metal ion and EDTA tested). In cases where no RNase activity was observed in the presence of one or more of the tested metal ions, all such data are presented together as a single red curve marked with red asterisks. The intensity of the 20 kDa RNase spot at 20 mM EDTA for the body wall and 23 kDa RNase spot at 1 mM MnCl_2_ for the gut was taken as 100% activity (Panels (**A**,**F**)), as it represented the highest value among the enzymes in this experiment. Results are shown as mean *±* SD from two experiments. All profiles differed significantly in positions of the maxima, amplitude, and shape of the curves (*p* < 0.0001, two-way ANOVA).

Figure 7 shows the dependence of RNase activity on the concentration of various metal ions and EDTA for RNase activities corresponding to protein complexes isolated from the body wall (at pH 5.5) and the gut (at pH 6.0).

As shown in Figure 7A–C, the highest RNase activity in the body wall complex was observed for the ~20 kDa enzyme at metal ion concentrations of 10–20 mM. This RNase(s) was activated by Zn^2+^ (5 mM) and, at 20 mM, by other metal ions in the following order: Mn^2+^ > Mg^2+^ ≈ Ca^2+^ > Co^2+^, whereas Ni^2+^ did not affect the activity of nucleases corresponding to this protein band. It is possible that these enzymes are effectively bound to certain metal ions, and therefore the addition of EDTA led to a very strong increase in their RNase activity (Figure 7A). Nucleases corresponding to the ~60 kDa protein band were noticeably activated by Mg^2+^ > Mn^2+^ and by EDTA; other metal ions did not have a considerable effect on the activity of the enzymes in this band. RNases of ~135 kDa, which are components of the body wall complex, were also activated by Mg^2+^ > Mn^2+^ ions at concentrations of 10–20 mM, but not by EDTA or other metal ions (Figure 7C).

Figure 7D–F present data on the metal dependence of ribonucleases from the gut protein complex. The highest metal-dependent activity was found for the ~20 kDa protein band. Nucleases in this band were activated by Mg^2+^ > Ca^2+^ > Co^2+^, as well as by EDTA (Figure 7D). Other metal ions did not activate these nucleases.

The enzyme corresponding to the ~48 kDa protein band was a Mg^2+^-dependent RNase (5 mM) and was activated to a much lesser extent by Mn^2+^ ions (5 mM) and EDTA (10–20 mM). As indicated above, the analysis of optimal pH values for protein bands after SDS-PAGE was carried out in the presence of 5 mM MgCl_2_. In this regard, the data in Figure 7F are of particular interest. In the absence of metal ions, the ~23 kDa protein band exhibited high activity. The addition of various metal ions led to suppression of this RNase activity in the following order: Zn^2+^ > Ni^2+^ > Co^2+^ > Mg^2+^. At low concentrations of approximately 1–2 mM, Mn^2+^ and Ca^2+^ slightly increased RNase activity, but increasing the concentration of these ions resulted in a strong decrease in activity. EDTA at low concentrations (1–3 mM) also increased RNase activity but decreased it at higher concentrations. It should be assumed that the ~23 kDa protein band of the gut complex corresponds to a metal-independent RNase; however, the same protein band may contain an admixture of a nuclease activated by Mg^2+^ and Ca^2+^ ions. Accordingly, at low concentrations of metal ions, activation of the contaminating metal-dependent nuclease occurs, whereas at higher concentrations, inhibition of the metal-independent enzyme takes place.

### 2.6. RNase Activity of Protein Complexes from Gonads

**Figure 8:**
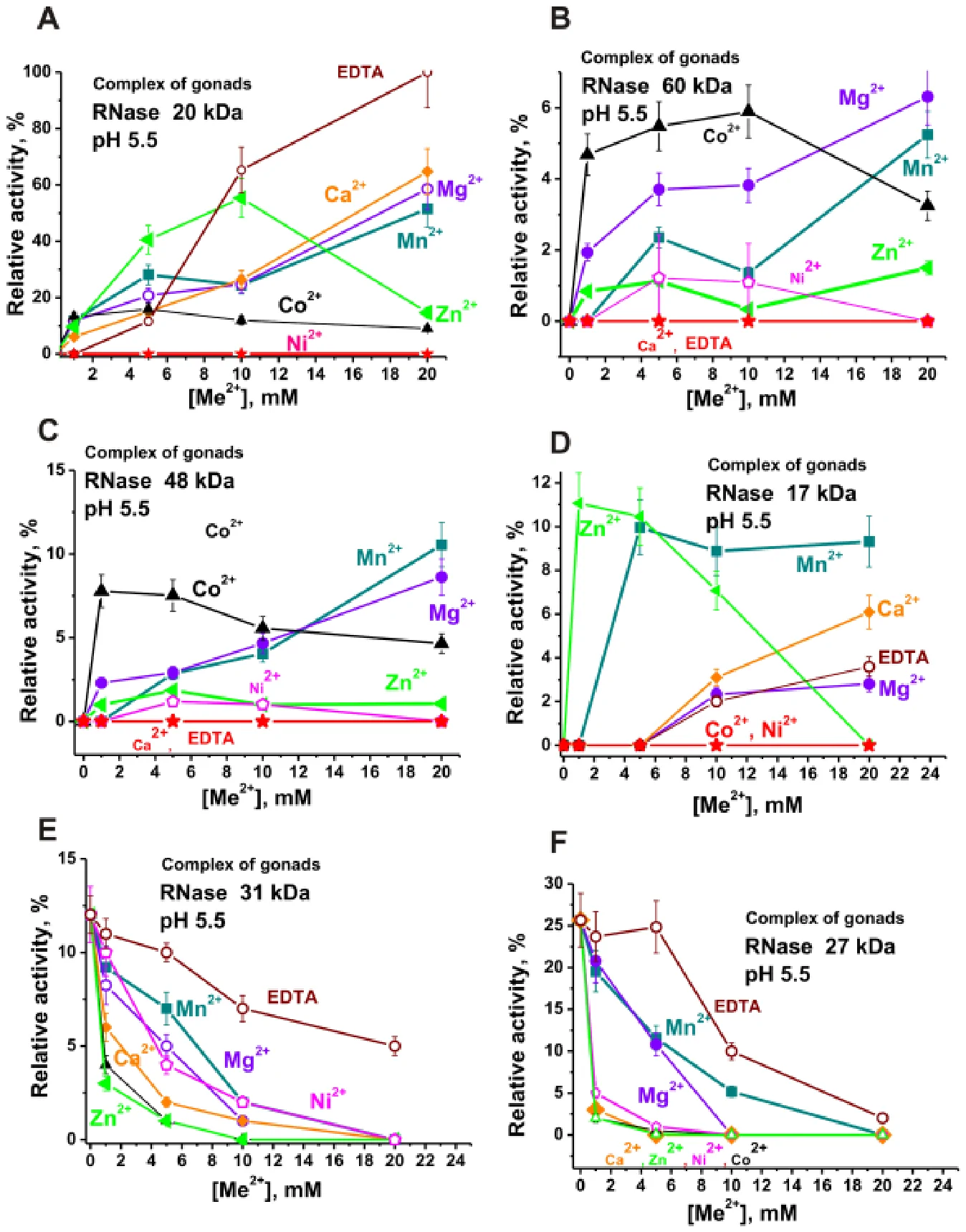
Dependence of relative RNase activity at pH 5.5 on the concentration of various metal ions and EDTA for enzymes of different molecular weights present within the protein complex of the gonads (kDa): 20 (**A**), 60 (**B**), 48 (**C**), 17 (**D**), 31 (**E**), and 27 (**F**). The data were obtained using in situ SDS-PAGE analysis (two gels for each concentration of every metal ion tested). In cases where no RNase activity was observed in the presence of one or more of the tested metal ions, all such data are presented together as a single red curve marked with red asterisks. The intensity of the 20 kDa RNase spot at 20 mM EDTA was taken as 100% activity (Panel (**A**)), as it represented the highest value among the enzymes in this experiment. Results are shown as mean *±* SD from two experiments. All profiles differed significantly in positions of the maxima, amplitude, and shape of the curves (*p* < 0.0001, two-way ANOVA).

Figure 8 shows the data on the dependence of RNase activity on the concentration of various metal ions and EDTA for enzymes present within the protein complex isolated from the gonads.

As in the case of the body wall complex (Figure 7), the maximum increase in RNase activity for the gonadal complex was observed for the ~20 kDa protein band (Figure 8A). The primary activators of these nucleases were Zn^2+^ (10 mM), as well as Mg^2+^, Ca^2+^, and Mn^2+^ ions (5–20 mM). Co^2+^ ions activated the enzyme to a much lesser extent, while Ni^2+^ ions did not noticeably affect activity. Notably, EDTA served as a very strong activator of this enzyme, which may indicate inhibition by certain metal ions associated with the complex.

Nucleases corresponding to the ~60 kDa protein band were inactive in the absence of metal ions (Figure 8B). The most effective activator of this gonadal complex enzyme was Co^2+^ (10 mM), with lower activity observed in the presence of other metal ions in the following order: Mg^2+^ > Mn^2+^ > Zn^2+^ ≈ Ni^2+^ (5–20 mM). Ca^2+^ ions and EDTA did not affect nuclease activity. Similar to the ~60 kDa nuclease, the ~48 kDa enzyme exhibited no activity in the absence of metal ions and was strongly activated by Co^2+^ ions (Figure 8C). Other metal ions also activated this RNase activity, albeit to a lesser extent: Mg^2+^ ≈ Mn^2+^ > Zn^2+^ ≈ Ni^2+^. Ca^2+^ ions and EDTA had no effect on enzyme activity.

Upon analyzing the dependence on various metal ion concentrations, a novel protein band of ~17 kDa was detected that exhibited RNase activity. At low concentrations, this ribonuclease was strongly activated by Zn^2+^ (1 mM) and Mn^2+^ (5 mM) ions (Figure 8D). Ca^2+^, Mg^2+^, and EDTA activated this nuclease at higher concentrations (10–20 mM), whereas Co^2+^ and Ni^2+^ ions did not affect enzyme activity. Of particular interest are the data on two nucleases from the gonadal complex that display metal-independent RNase activity. One nuclease has a molecular weight of ~31 kDa (Figure 8E), and the second of ~27 kDa (Figure 8F). Both nucleases demonstrated high RNase activity in the absence of metal ions. Various metal ions inhibited the activity of these enzymes in slightly different manners. The degree of activity suppression for the ~31 kDa nuclease decreased in the following order: Zn^2+^ ≈ Co^2+^ ≈ Ca^2+^ ≥ Ni^2+^ ≈ Mg^2+^ ≈ Mn^2+^. EDTA also inhibited this enzyme, though to a lesser extent (Figure 8E). A nearly identical pattern was observed for the ~27 kDa nuclease: Zn^2+^ ≈ Co^2+^ ≈ Ca^2+^ ≈ Ni^2+^ ≈ Mg^2+^ ≥ Mn^2+^ (Figure 8F). At low concentrations (1–5 mM), EDTA did not alter enzyme activity, but increasing its concentration led to a decrease in activity.

Due to the limited amount of protein complex material obtained from the respiratory trees, the metal ion dependence of RNases from this organ was not analyzed. Data on RNases present in complexes from four organs and their dependence on metal ions are summarized in Table 2.

Table 2 demonstrates that stable protein complexes isolated from different organs—coelomic fluid (13), gonads (6), body wall (3), and gut (3)—contain variable numbers of metal ion-dependent and metal-independent RNases under the selected experimental conditions. Among these RNase activities, some are dependent on a single type of metal ion, while others require several different metal ions for activity. The optimal concentrations of these metals range from 1.0 to 20 mM, and certain metal ions activate RNase activities across a broad concentration range.

The unified inventory of all detected bands and the conditions of their detection is provided in Table 3.

Given the data on the dependence of RNase activities on optimal pH values and the presence of various metal ions, it remains challenging to draw definitive conclusions regarding the total number of RNases present in complexes from different organs. This difficulty arises because some enzymes with RNase activity can be activated not only by a primary metal ion, such as magnesium, but also by other metal ions, albeit to a lesser extent. A similar phenomenon has been observed for DNase I, where magnesium ions serve as the primary cofactor, yet other metal ions can also contribute to enzyme activation, though less efficiently [64]. The same pattern holds for certain RNases, for which one metal ion acts as the primary activator [3,26], while other metal ions retain the capacity to activate these nucleases to a lesser degree.

## 3. Discussion

The functional characteristics of numerous RNases in humans and other animals have been described in the literature [1,2,3,4,5,6,7,8,9,10,11,12,13,14,15,16,17,18,19,20,21,22,23,24,25,26], and several review articles have been devoted to this topic [3,4,5,6,7,24,25,26]. Among them, the best studied is bovine pancreatic RNase A (13.7 kDa), which cleaves RNA specifically after pyrimidine nucleotides [6,7,8,9]. Other mammalian pyrimidine-specific RNases have pH optima in the range of 6.5–8.0, hydrolyze poly(U) faster than poly(C), and are inhibited by Cu^2+^ and Zn^2+^ [10]. Additional ribonucleases that preferentially cleave RNA after cytidine residues have also been described [3,4,5,6,7]. Some microbial RNases (approximately 12 kDa) exhibit pH optima between 7 and 8 and show specificity for guanylic acid [23]. Echinoderm RNases belong to the T2 type, have small molecular masses of 15–77 kDa, and predominantly acidic to neutral pH optima (5–7) [28,29,30,31,32].

The combined properties of the sets of RNA-hydrolyzing activities detected in preparations of the highly stable complexes isolated from various organs of sea cucumbers do not generally correspond to the properties of the RNases described above. Among these enzymes, both metal-independent and metal-dependent RNase activities are present, exhibiting at least two optimal pH values and various optimal concentrations of diverse metal ions (Table 1 and Table 2). However, a subset of these enzymes shares similarities with the described echinoderm RNases. These are the active protein bands with masses of approximately 48 kDa in coelomic fluid, ~31 and 27 kDa in gonads, ~23 kDa in all organs except gonads, ~25 kDa in all organs except the body wall, and ~20 kDa in the body wall and respiratory trees (Figure 2 and Figure 3). These RNases demonstrated a pH optimum only at around pH 6.0 and have relatively low molecular masses. Nevertheless, all data regarding the analysis of RNases in various complexes from different sea cucumber organs should be interpreted in conjunction with previously obtained data on these complexes [39,40,41,42,43,44].

It is reasonable to assume that many specific biological functions of various RNases remain to be discovered. It is well established that the activity of certain RNases leading to RNA degradation is essential for a wide range of normal physiological processes and for organismal survival [1,2,3,4,5,6,7,8,9,10,11,12,13,14,15,16,17,18,19,20,21,22,23,24,25,26]. Consequently, elucidating the diverse functions of these enzymes is a necessary and active area of current research. The available data to date suggests that echinoderm RNases, including those of holothurians, may play important roles in several key physiological processes beyond simple RNA degradation. Involvement in regeneration is one of the most likely functions, given that during regeneration in *E. fraudatrix*, enhanced expression of genes encoding proteins with RNase activity (e.g., PIWI) has been observed [37]. In addition to regenerative processes, RNases may influence fundamental cellular processes such as proliferation, migration, and signal transduction. The ability of RNases to selectively hydrolyze RNA allows them to fine-tune the levels of specific transcripts, thereby participating in the regulation of numerous cellular responses. Furthermore, the presence in sea urchins of a T2-type RNase whose activity sharply changes in gonads depending on the stage of maturity points to a possible role of RNases in sex determination or gamete maturation processes in echinoderms. The presented data indicate that echinoderm RNases, including the recently characterized enzymes from *E. fraudatrix*, are not merely degradative enzymes but rather multifunctional molecules potentially involved in a wide range of biological processes, from regeneration to immunity. Moreover, considering that these enzymes may operate within high-molecular-weight protein complexes, their functional potential is significantly enhanced: functioning within a multiprotein ensemble not only allows for increased catalytic efficiency through cooperative interactions but also enables integration of RNase activity with other enzymatic functions (e.g., proteolysis or RNA degradation) within a single molecular machine. In addition, association of RNases with other complex components may modulate their substrate specificity and stability, opening additional possibilities for fine-tuning cellular responses in different tissues and at different stages of regenerative morphogenesis. The observation that the RNase composition of the complexes differed among organs is consistent with these suggestions.

Furthermore, it should be noted that, based on the data obtained thus far, it cannot yet be concluded that all of the RNase activities we detected within the stable complexes correspond to individual RNases. Based on the combinations of apparent molecular weights (~10, 20, 23, 25, 44, 48, 60, 100, 135, 180 kDa) and optimal pH values (4.0–9.5), it can be inferred that approximately more than 20–25 distinct forms of RNase activity are present overall in five stable complexes of the holothurian *P. chilensis*. It should be noted that canonical RNase A, T1, and T2 family enzymes are generally metal-independent. Therefore, the metal-dependent RNA-hydrolyzing activities observed in this study may not belong to these families and could represent other classes of nucleases (e.g., metal-dependent endo- or exonucleases) or RNases with non-canonical metal dependence. Thus, the terms “RNase” and “RNase activity” are used here operationally to denote RNA-hydrolyzing activities, not necessarily canonical RNases.

Several limitations of this study should be noted. First, all experiments were performed using pooled organ samples from 30–40 animals, and although technical replicates were included (two independent gels for each condition), biological replicates were not performed. Second, in situ zymography, although effective for detecting multiple RNase forms in a complex mixture, does not allow unambiguous assignment of individual bands to a single enzyme species, as co-migrating proteins with similar molecular weights and overlapping pH/metal dependencies may contribute to the same band. Some RNase activities with similar or slightly different molecular weights may additionally correspond to different RNases or to the same enzyme that has undergone glycosylation, phosphorylation, or other post-translational modifications. Therefore, the estimated number of different RNases per organ (e.g., 13 in coelomic fluid; Table 3) should be considered as a provisional estimate rather than a definitive number. Third, although we hypothesize that these RNase assemblies may be involved in regeneration based on the known involvement of RNases in the regulation of gene expression and the high regenerative capacity of holothurians, we did not directly assess RNase activity or complex composition during regenerative processes (e.g., after evisceration) or correlate the observed enzymatic profiles with specific stages of tissue repair. Therefore, any functional relationship between the identified RNases and regeneration remains, at present, speculative. Fourth, the metal ion dependence of RNases was not analyzed for the respiratory tree complex due to the insufficient amount of material. Therefore, the data on metal activation for this organ are incomplete, and the corresponding column is absent from Table 2. Fifth, the apparent number of RNase bands differed between the pH-dependence experiments (5.0 mM MgCl_2_) and the metal-dependence experiments (various metal ions and concentrations). This is an inherent limitation of the in situ zymography approach: the detection of individual RNases depends on the activation conditions, and bands with low activity under one set of conditions may become detectable under another. Therefore, the band counts reported in Table 1, Table 2 and Table 3 should be considered condition-specific rather than absolute. In addition, the pH-dependence and metal-dependence series were performed on independently prepared gels with different normalization references; relative band intensities are not quantitatively comparable between these series. Sixthly, the pH screening was performed using four different buffers; therefore, the apparent multiple pH optima observed for some bands may partly reflect buffer effects. In particular, pH 7.0 was buffered with Tris-HCl, which has low buffering capacity at this pH; therefore, the apparent activity minimum at pH 7.0 may partly reflect inadequate buffering rather than a genuine property of the enzymes, whereas the adjacent pH 6.5 point was covered by MES-NaOH. In addition, all pH profiles were obtained in the presence of 5.0 mM MgCl_2_, which may mask the intrinsic pH optima of metal-independent or Mg^2+^-inhibited activities.

In addition, we investigated RNase activities in complexes from only four organs and the coelomic fluid. Considering that we did not study RNase activities in stable complexes of all organs of the sea cucumber, there may be more of them. It is possible that other RNases with different molecular weights and optimal pH values are present in other organs of the sea cucumber. For example, some organs may contain RNases activated by other metal ions. Taking this into account, such multifunctional complexes are of particular interest for future research. Therefore, further studies are necessary for a complete understanding of the role of RNases within protein complexes in the physiology of these unique marine animals.

The data obtained in this study on ribonuclease assemblies within highly stable multiprotein complexes of sea cucumbers are of not only fundamental but also significant applied significance, opening up prospects for aquaculture and biomedicine. In the case of sea cucumbers, which have been used for centuries in traditional Asian medicine as a valuable tonic, their bioactive components, including peptides and enzymes, exhibit a wide range of pharmacological effects—immunomodulatory, antibacterial, antiviral, anti-inflammatory, and antitumor [65]. In this context, the RNase activities we detected in stable complex preparations could potentially contribute to the immune defense of sea cucumbers by participating in the degradation of foreign RNA from pathogens and the fine regulation of inflammatory responses. Furthermore, the ability of RNases to modulate gene expression and RNA processing suggests a possible role in immunoregulation, consistent with the known immunomodulatory properties of bioactive compounds from holothurians. Of particular interest are potential applications in aquaculture, where stress, injury, and infectious diseases are major factors reducing productivity. Biologically active compounds from holothurians, including RNases, may be used to accelerate wound healing, enhance stress resistance, and enhance the immune response in cultured aquatic organisms. Extracts and hydrolysates of holothurian tissues have already demonstrated efficacy in accelerated wound healing models, and it is likely that RNases, along with other components, contribute to these effects.

## 4. Materials and Methods

### 4.1. Reagents and Samples

Most compounds used in this study were obtained from Sigma (St. Louis, MO, USA). Superdex 200 HR 10/30 and Sepharose 4B columns were purchased from GE Healthcare Life Sciences (Marlborough, MA, USA). Adult sea cucumbers *Paracaudina chilensis* were collected in September 2025, as described previously [39] from Peter the Great Bay, Sea of Japan. A description of the habitat conditions of sea cucumbers in Peter the Great Bay of the Sea of Japan is provided in [39]. All sea cucumber samples and organs obtained were frozen and stored at –40 °C prior to experimentation. Mixtures of pooled samples from various organs of 30–40 sea cucumbers (10–20 g) were used. The methods for preparing homogenates from various organs and for isolating stable complexes from the individual organs of the sea cucumber have been described in detail elsewhere [39]. The average molecular weights of the five complexes obtained previously [39] were different (in megaDaltons): 1.2 ± 0.2 for coelomic fluid; 1.4 ± 0.1 for respiratory trees; 1.5 ± 0.1 for gut; 1.7 ± 0.2 for gonads; and 1.7 ± 0.2 for body wall (Figure S1) [39]. Protein concentration was determined by the Bradford method using bovine serum albumin as a standard. Absorbance was measured at 595 nm, and protein concentrations were calculated from a standard curve. All of these complexes were used for the analysis of their RNase activity. This study utilized sea cucumbers, which are marine animals, and ethical approval is not currently required for work with fish and other marine and oceanic organisms, including sea cucumbers. The Institute of Chemical Biology and Fundamental Medicine of the Siberian Branch of the Russian Academy of Sciences supported our work with marine organisms.

### 4.2. In Situ Analysis of RNase Activity

For the analysis of RNase activity in stable protein complexes from different organs, the in situ SDS-PAGE method was employed. In situ analysis of RNase activity was performed as described previously [60,61,62]. The complexes (4 µg) were incubated at 23 °C for 20 min in 20 mM Tris-HCl (pH 7.5) containing 0.1% SDS and subjected to 5–18% SDS-PAGE using gels containing 40 μg/mL of polymeric yeast RNA, which was added to the solution prior to gel polymerization. The gels were then washed with a solution of 4.0 M urea and rinsed four times with water to remove SDS and allow RNase renaturation. For cleavage of polymeric RNA, the gels were first incubated for 24 h at 37 °C in solutions containing 5.0 mM MgCl_2_ and 20 mM different buffers with pH values in the range of 3.0–10.0: 3.0, 3.5, 4.0, 4.5, 5.0, 5.5, 6.0, 6.5, 7.0, 7.5, 8.0, 8.5, 9.0, 9.5, and 10.0. To obtain buffers at various pH values, the following buffers were used: sodium acetate buffer (pH 3.0–5.0), MES-NaOH (pH 5.3–6.6), Tris-HCl (pH 7.0–8.6), and glycine-NaOH (pH 9.0–10.0). Thus, pH 6.0 and 6.5 were buffered with MES-NaOH, and pH 7.0–8.6 with Tris-HCl.

When analyzing the optimal concentrations of various metal ions and EDTA at different pH values, these were added to the reaction mixtures at concentrations ranging from 0 to 20 mM. For each pH value and metal ion and EDTA concentration, two to three independent experiments were performed for each complex preparation. To detect the regions of polymeric RNA hydrolysis by the various RNases present in the complexes, the gels were subsequently stained with 0.5 µg/mL ethidium bromide. Original gel images after staining with ethidium bromide are provided in Supplementary Figure S3.

The positions of enzyme-dependent RNA hydrolysis were visualized as dark spots, corresponding to the removal of short RNA fragments during prior gel processing and thus indicating an absence of stained RNA against the background of a fluorescent gel. Parallel longitudinal gel slices were used to determine the positions of different proteins by standard Coomassie Blue staining (Supplementary Figure S4). To determine the molecular weights of the RNases on the gels, a calibration curve was constructed using control proteins of known molecular weights.

### 4.3. Gel Visualization and Densitometric Analysis

Gels were documented using the Gel Doc XR+ imaging system (Bio-Rad Laboratories, Hercules, CA, USA) running Image Lab software (version 6.1). The system is equipped with a CCD camera featuring a pixel size of 4.65 × 4.65 µm and 12-bit resolution (4096 gray levels), providing a dynamic range exceeding three orders of magnitude. For ethidium bromide-stained gels, images were captured under UV transillumination. For Coomassie-stained gels, images were captured in colorimetric mode with epi-white illumination. Non-uniform lighting was corrected automatically via the Dynamic Flat Fielding function, and all raw images were stored as 16-bit TIFF files to preserve full dynamic range for subsequent densitometric analysis.

Quantitative evaluation of zymograms was carried out using Image Lab software (version 6.1). Zymograms represent negative images: zones of RNA hydrolysis appear as dark bands against a fluorescent background. Data inversion was therefore applied prior to densitometric analysis. Background signal was subtracted using the local background subtraction algorithm. For each detected band, the software automatically computed the Adjusted Volume, representing the integrated optical density after background correction. A detection threshold of 0.5% of the maximum signal (approximately 600,000 arbitrary units) was applied; signals falling below this threshold were discarded as background noise. To normalize the data, the highest band intensity observed across the entire experimental series was defined as 100% activity.

### 4.4. Statistical Analysis

All experiments were performed in two independent technical replicates. Data are presented as mean ± standard deviation (SD). Values falling below the limit of detection (signal less than 1/100 of the maximum mean activity for each compound) were replaced with conservative upper bounds equal to 1/100 of the maximum activity (limits ranged from 0.01 to 1.0 depending on the dataset). For such points, the SD was set to 0.01 to provide a deliberately conservative estimate of significance; sensitivity analysis confirmed that varying these limits or assigned SDs did not alter the conclusions. Rounding of means to 0.1 did not introduce systematic error. Comparison of activity profiles was performed using two-way analysis of variance (ANOVA) with interaction. For pH-dependence experiments, factors were “compound” and “pH”; for concentration-dependence experiments, factors were “compound” and “concentration”. A sensitivity analysis, in which zero activities were varied from zero to the corresponding detection limits, did not alter the conclusion regarding the significance of differences between the profiles. All calculations were performed in the R environment (version 4.3.1).

## 5. Conclusions

In this work, we provide the first evidence that highly stable multiprotein complexes from different organs of the sea cucumber *Paracaudina chilensis* contain repertoires of ribonuclease activities with substantially different biochemical properties. Using in situ zymography, we identified 13 distinct RNA-hydrolyzing activities (apparent-MW bands) in the coelomic fluid complex, 8 in gonads, 6 each in gut and respiratory trees, and 5 in the body wall complex (Table 3); the individual enzymes responsible for these activities remain unidentified. Many RNase-positive bands showed pH optima in more than one range, suggesting the presence of multiple active sites or co-migrating enzymes with similar molecular weights. It should be noted, however, that all pH profiles were obtained in the presence of 5.0 mM MgCl_2_, and the pH screen was performed using four different buffers without overlap controls. Therefore, the apparent pH optima reported here should be considered provisional and may be influenced by buffer composition and by Mg^2+^ binding. A wide variability in metal dependence was observed: some enzymes were activated by Mg^2+^, others by Ca^2+^, Mn^2+^, Co^2+^, Zn^2+^, or Ni^2+^, with optimal concentrations ranging from 1 to 20 mM. Several activities were also enhanced by EDTA, which may reflect chelation of divalent cations from the assay environment and/or from the complexes; however, the source of these cations was not determined in the present study. The metal ion dependence of RNases from the respiratory tree complex remains uncharacterized due to limited material; future studies with larger sample amounts will be needed to complete this analysis. Comparison across organs revealed that even protein bands with identical molecular weights often differed in pH optima and metal responsiveness, which may indicate the presence of isoenzymes that differ among tissues or of post-translational modifications. Our results expand the current understanding of RNase activities in echinoderms and suggest their involvement in regeneration, immunity, and cellular metabolism. Future studies should focus on identifying the individual enzymes (e.g., by LC-MS/MS), elucidating whether the detected enzymes are physically associated with the complexes and which mechanisms govern the organ-specific composition of these activity sets, identifying their substrate specificities, and evaluating their possible contribution to regenerative processes in sea cucumbers.

## Figures and Tables

**Table 1:** Data on the optimal pH values and relative RNase activities corresponding to highly stable complexes from different organs of the sea cucumber.

Apparent MW of RNase, kDa	Optimal pHs (Relative Activities, % in Comparison with RNase with Apparent MW ≈ 23 kDa)									
	Coelomic Fluid		Body Wall		Gut		Gonads		Respiratory Trees	
	Optimal pH	Relative Activity, %	Optimal pH **	Relative Activity, %	Optimal pH	Relative Activity, %	Optimal pH	Relative Activity, %	Optimal pH	Relative Activity, %
~20	5.5	12.5	4.0–4.5	1.9	4.5–5.5	11.4	5.5	3.1	4.0	9.4
	8.5	3.7	5.5	4.2	6.5	4.9	9.0	1.0	5.0	6.6
	-	-	-	-	8.5	15.9	-	-	6.5	5.5
~23	6.0	100 *	4.0–4.5	2.7	4.5	18.3	-	-	4.5	1.6
	-	-	6.0–6.5	4.2	6.0	91.1	-	-	6.0	10.0
~25	5.5	10	-	-	5.0	20.0	4.0–5.0	6.4	4.5–5.5	28.6
~27	-	-	-	-	-	-	4.5–5.0	4.9	-	-
~31	-	-	-	-	-	-	4.5–5.0	2.9	-	-
~44	6.0	35	-	-	-	-	-	-	-	-
	9.0	61.2	-	-	-	-	-	-	-	-
~48	5.5	16.4	7.5	2.9	6.0	38.7	7.5	5.9	6.0	4.8
	-	-	8.0–9.0	2.1	8.0–9.0	70.1	8.0–9.0	4.4	7.5	13.4
	-	-	-	-	-	-	-	-	9.0	8.1
~60	5.5–6.6	37.1	6.5	6.1	6.5–7.0	17.3	6.5	3.7	5.5	2.6
	9.0	51.1	8.5–9.0	6.8	8.0	14.7	8.5	7.1	8.5	8.5
	-	-	-	-	9.5	14.6	-	-	-	-
~100	6.0	4.4	-	-	-	-	-	-	-	-
	9.0	9.4	-	-	-	-	-	-	-	-
~135	5.5–6.0	32.3	6.5	6.1	6.0	26.7	6.0	2.7	6.0	5.9
	8.0–9.0	57.4	7.5	6.9	9.0	65.3	7.5–8.0	6.9	7.5	10.7
			8.5–9.0	8.3			9.0	8.6	9.5	11.7

* All RNase activities of stable protein complexes from different organs are expressed in relative units compared to the activity of the RNase with a molecular weight of ~23 kDa from the coelomic fluid complex, which was set at 100%. ** Where an extended optimal pH range was observed, clearly indicating two distinct optima, the values are presented as a range, e.g., 4.0–4.5 or 8.0–9.0.

**Table 2:** Combined data on various RNases in the stable complexes from different organs.

Apparent MW of RNase, kDa	Apparent MWs of Protein Bands Demonstrating RNase Activity and Effect of Metal Ions							
	Coelomic Fluid		Body Wall		Gut		Gonads	
	Number of RNases							
	13		3		3		6	
~10	Activator (mM)	Inhibitor ^µ^	Activator (mM)	inhibitor	Activator (mM)	Inhibitor	Activator (mM)	Inhibitor
	Mg^2+^ (1–20) *	Ni^2+^, Co^2+ µ^	-	-	-	-	-	-
	Mn^2+^ (10) *		-	-	-	-	-	-
	Zn^2+^ (10)		-	-	-	-	-	-
	Ca^2+^ (20)		-	-	-	-	-	-
	EDTA		-	-	-	-	-	-
~15	Mg^2+^ (1)	Ca^2+^, EDTA	-	-	-	-	-	-
~17	EDTA	Ca^2+^	-	-	-	-	Zn^2+^ (1) **	-
	-	-	-	-	-	-	Mn^2+^ (5)	-
	-	-	-	-	-	-	Ca^2+^ (10–20)	-
	-	-	-	-	-	-	Mg^2+^ (10–20)	-
	-	-	-	-	-	-	EDTA	-
~20	Mg^2+^ (10–20)	Ca^2+^, Mn^2+^, Ni^2+^, Co^2+^, EDTA	Zn^2+^ (5)	-	Mg^2+^ (20)	-	Zn^2+^ (10)	-
	Zn^2+^ (5)		Mn^2+^ (20)	-	Ca^2 +^ (10)	-	Mn^2+^ (5–20)	-
			Mg^2+^ (20)	-	Mn^2+^ (20)	-	Mg^2+^ (5–20)	-
			Ca^2+^ (10)	-	EDTA	-	Ca^2+^ (5–20)	-
			Co^2+^ (20)	-		-	Co^2+^ (1–5)	-
			EDTA	-		-	EDTA	-
~23	Mg^2+^ (1–4)	Ca^2+^, Mn^2+^ Ni^2+^, Co^2+^, Zn^2+^, EDTA	-	-	Ca^2+^ (1),	Zn^2+^, Ni^2+^, Co^2+^, Mg^2+^	-	-
					Mn^2+^ (1)		-	-
~25	Ca^2+^ (10)	Mn^2+^, Ni^2+^, Co^2+^, Zn^2+^, EDTA	-	-	-	-	-	-
			-	-	-	-	-	-
			-	-	-	-	-	-
~27	-	-	-	-	-	-		Zn^2+^, Co^2+^ Ca^2+^, Ni^2+^ Mg^2+^, Mn^2+^, EDTA
	-	-	-	-	-	-		
	-	-	-	-	-	-		
	-	-	-	-	-	-		
~31	-	-	-	-	-	-		Zn^2+^, Co^2+^, Ca^2+^, Ni^2+^, Mg^2+^, Mn^2+^, EDTA
	-	-	-	-	-	-	-	
	-	-	-	-	-	-	-	
	-	-	-	-	-	-	-	
~42	Mg^2+^ (1–20)	Ca^2+^, EDTA	-	-	-	-	-	-
~44	Mg^2+^ (1–20)	Ni^2+^, Zn^2+^,	-	-	-	-	-	-
	Mn^2+^ (5–10)		-	-	-	-	-	-
	Co^2+^ (10),		-	-	-	-	-	-
	Ca^2+^ (5)		-	-	-	-	-	-
~48	Mg^2+^ (5–20)	-	-	-	Mg^2+^ (5)	-	Co^2+^ (1)	-
	Mn^2+^ (1)				Mn^2+^ (5)		Mg^2+^	
	Co^2+^ (1)	-	-	-	EDTA	-	Mn^2+^	-
	Ca^2+^ (1)	-	-	-		-	Zn^2+^	-
	Zn^2+^ (1)	-	-	-		-	Ni^2+^	
	Ni^2+^ (1–2).	-	-	-		-		-
	EDTA	-	-	-		-		-
~60	Mg^2+^ (5–20)		Mg^2+^ (5–20)	-	-	-	Co^2+^ (10 mM)	-
	Mn^2+^ (5)		Mn^2+^ (5–20)	-	-	-	Mg^2+^ (5–20)	-
	Co^2+^ (10)		EDTA	-	-	-	Mn^2+^ (5–20)	-
	Ca^2+^ (10)		-	-	-	-	Zn^2+^ (5–20)	-
	Zn^2+^ (10)		-	-	-	-	Ni^2+^ (5–20)	-
	Ni^2+^ (5)		-	-	-	-		-
~100	Mn^2+^ (5–20)	Ca^2+^, Zn^2+^, Ni^2+^	-	-	-	-	-	-
	Mg^2+^ (1–20)		-	-	-	-	-	-
	Co^2+^ (1)		-	-	-	-	-	-
~135	Co^2+^ (1)	Ca^2+^, Zn^2+^, Ni^2+^	Mg^2+^ (10–20)	-	-	-	-	-
	Mn^2+^ (10)		Mn^2+^ (10–20)	-	-	-	-	-
	Mg^2+^ (1–20)		-	-	-	-	-	-
~180	Mg^2+^ (1–20)	-	-	-	-	-	-	-
	Co^2+^ (1)	-	-	-	-	-	-	-

* Some nucleases of the protein complexes exhibit a clearly defined optimal concentration of metal ions, which is indicated in parentheses as a single value. In cases where a relatively wide range of metal ion concentrations supports activity, the range is provided (e.g., 5–20 mM). ** The activator columns list metal ions that activate the enzyme; activation efficiency increases from left to right (from lower to higher activity). ^µ^ The inhibitor columns list metal ions that suppress nuclease activity. If no metal ions are listed in this column, this indicates that the tested metal ions had no effect on enzyme activity. In the case of several metal-independent RNases, activation by EDTA was observed. The number of RNases listed in this table was determined under metal-dependence conditions (various metal ions at 0–20 mM) and therefore differs from the band counts in Table 1, which were obtained with 5.0 mM MgCl_2_ only. The metal ion dependence of RNases from the respiratory tree complex was not analyzed due to insufficient material.

**Table 3:** Unified inventory of RNA-hydrolyzing activity bands (apparent MW) detected in the stable multiprotein complexes from different organs of *P. chilensis*.

Apparent MW of RNase, kDa	Coelomic Fluid	Body Wall	Gut	Gonads	Respiratory Trees	Detected in
**~10**	+ *	–	–	–	–	metal series, pH 5.5
**~15**	+	–	–	–	–	metal series, pH 8.5
**~17**	+	–	–	+	–	metal series (pH 8.5/pH 5.5)
**~20**	+	+	+	+	+	both series
**~23**	+	+	+	–	+	both series (except for the body wall in the metal series)
**~25**	+	–	+	+	+	pH series; both series for coelomic fluid
**~27**	–	–	–	+	–	both series
**~31**	–	–	–	+	–	both series
**~42**	+	–	–	–	–	metal series, pH 8.5
**~44**	+	–	–	–	–	both series
**~48**	+	+	+	+	+	both series (except for the body wall in the metal series)
**~60**	+	+	+	+	+	both series (except for the gut in the metal series)
**~100**	+	–	–	–	–	both series (except for pH 8.5)
**~135**	+	+	+	+	+	both series (except for the gut and gonads in the metal series, and for the coelomic fluid at pH 8.5)
**~180**	+	–	–	–	–	metal series, pH 5.5
Union count **	**13**	**5**	**6**	**8**	**6**	

* “+”, band detected under at least one set of conditions; “–”, not detected. Detection conditions (last column): the pH-dependence series (5.0 mM MgCl_2_; Section 2.3, Table 1), the metal-dependence series at pH 5.5, and the metal-dependence series at pH 8.5 (Section 2.4). ** Including five bands (~10, ~15, ~17, ~42 and ~180 kDa) detected only in the metal-dependence series. The metal-dependence series was not performed for the respiratory trees due to insufficient material; for this organ, detection refers to the pH-dependence series only.

## Data Availability

The data that supports the findings of this study are available within the article and in Supplementary Figure S1 (obtaining of the complex using gel filtration on the Sepharose 4B), Supplementary Figure S2 (transmission electron microscope data of different stable complexes) and Supplementary Figures S3 and S4 (gels stained with ethidium bromide and Coomassie). Supplementary Figures S5 and S6—control experiments. Supplementary Table S1—raw, un-normalized integrated intensities of the ~23 and ~48 kDa RNase bands of the coelomic fluid complex. In addition, Supplementary Methods S1 and Supplementary Tables S1 and S2.

## Supplementary Materials

The following supporting information can be downloaded at: https://www.mdpi.com/article/10.3390/ijms272110051/s1.

## Abbreviations

DTT, dithiothreitol; EDTA, ethylenediaminetetraacetic acid; kDa, kilodaltons; MDa megadaltons; MW, molecular weight; SDS, sodium dodecyl sulfate; SDS-PAGE, sodium dodecyl sulfate-polyacrylamide gel electrophoresis; MALDI-TOF mass spectrometry—matrix-assisted laser desorption ionization mass spectrometry.

## References

[1] Shlyakhovenko V.O., Samoylenko O.A. (2019). The dual role of ribonucleases in tumor-host relationship. Exp. Oncol..

[2] Hameş E.E., Demir T. (2015). Microbial ribonucleases (RNases): Production and application potential. World J. Microbiol. Biotechnol..

[3] Becknell B., Spencer J.D. (2016). A Review of Ribonuclease 7’s Structure, Regulation, and Contributions to Host Defense. Int. J. Mol. Sci..

[4] Fang E.F., Ng T.B. (2011). Ribonucleases of different origins with a wide spectrum of medicinal applications. Biochim. Biophys. Acta.

[5] Sorrentino S. (2010). The eight human “canonical” ribonucleases: Molecular diversity, catalytic properties, and special biological actions of the enzyme proteins. FEBS Lett..

[6] Sorrentino S. (1998). Human extracellular ribonucleases: Multiplicity, molecular diversity and catalytic properties of the major RNase types. Cell Mol. Life Sci..

[7] Vlassov A.V. (1998). Human ribonucleases. Biochemistry.

[8] Liu Y., Xu Z. (2023). The Ribonuclease A Superfamily: Not Only a Class of Enzymes for RNA Degradation. Chin. J. Biochem. Mol. Biol..

[9] Kemp C., Imler J. (2009). Antiviral immunity in drosophila. Curr. Opin. Immunol..

[10] Nguyen T.L., Nguyen T.D., Ngo M.K., Le T.N., Nguyen T.A. (2010). T2 Family Ribonucleases: Ancient enzymes with diverse roles. Trends Biochem. Sci..

[11] Luhtala N., Parker R. (2023). Noncanonical processing by animal Microprocessor. Mol. Cell..

[12] Blackburn P., Moore S. (1982). The Enzymes.

[13] Mizuta K., Awazu S., Yasuda T., Kishi K. (1990). Purification and characterization of three ribonucleases from human kidney: Comparison with urine ribonucleases. Arch. Biochem. Biophys..

[14] Sorrentino S., Libonati M. (1997). Structure-function relationships in human ribonucleases: Main distinctive features of the major RNase types. FEBS Lett..

[15] Irie M., Nitta R., Ohgi K., Niwata Y., Watanabe H., Iwama M., Beintema J.J., Sanda A., Takizawa Y. (1988). Primary structure of a non-secretory ribonuclease from bovine kidney. J. Biochem..

[16] Beintema J.J., Wietzes P., Weickmann J.L., Glitz D.G. (1984). The amino acid sequence of human pancreatic ribonuclease. Anal. Biochem..

[17] Hofsteenge J., Matthies R., Stone S.R. (1989). Primary structure of a ribonuclease from porcine liver, a new member of the ribonuclease superfamily. Biochemistry.

[18] Vicentini A.M., Hemmings B.A., Hofsteenge J. (1994). Residues 36–42 of liver RNase PL3 contribute to its uridine-preferring substrate specificity. Cloning of the cDNA and site-directed mutagenesis studies. Protein Sci..

[19] Shapiro R., Fett J.W., Strydom D.J., Vallee B.L. (1986). Isolation and characterization of a human colon carcinoma-secreted enzyme with pancreatic ribonuclease-like activity. Biochemistry.

[20] Zhou H.M., Strydom D.J. (1993). The amino acid sequence of human ribonuclease 4, a highly conserved ribonuclease that cleaves specifically on the 3’ side of uridine. Eur. J. Biochem..

[21] Sorrentino S., Glitz D.G., Hamann K.J., Loegering D.A., Checkel J.L., Gleich G.J. (1992). Eosinophil-derived neurotoxin and human liver ribonuclease. Identity of structure and linkage of neurotoxicity to nuclease activity. J. Biol. Chem..

[22] Weickmann J.L., Elson M., Glitz D.G. (1981). Purification and characterization of human pancreatic ribonuclease. Biochemistry.

[23] Sorrentino S., Libonati M. (1994). Human pancreatic-type and nonpancreatic-type ribonucleases: A direct side-by-side comparison of their catalytic properties. Arch. Biochem. Biophys..

[24] Deshpande R.A., Shankar V. (2002). Ribonucleases from T2 family. Crit. Rev. Microbiol..

[25] Pizzo E., D’Alessio G. (2007). The success of the RNase scaffold in the advance of biosciences and in evolution. Gene.

[26] Durand S., Condon C. (2018). RNases and Helicases in Gram-Positive Bacteria. Microbiol. Spectr..

[27] Barnard E.A. (1969). Ribonucleases. Annu. Rev. Biochem..

[28] Motoyoshi N., Kobayashi H., Itagaki T., Inokuchi N. (2016). Amino acid sequence analysis and characterization of a ribonuclease from starfish *Asterias amurensis*. J. Biochem..

[29] Fernlund P., Josefsson L. (1966). Purification and characterization of ribonuclease from unfertilized eggs of the sea urchin, *Psammechinus miliaris*. Nature.

[30] Fernlund P., Josefsson L. (1968). Preparation and properties of sea-urchin ribonuclease. Biochim. Biophys. Acta.

[31] Baltus E. (1962). Étude de la ribonucléase des nucléoles isolés a partir d’oocytes d’astéries (*Asterias rubens*). Biochim. Biophys. Acta.

[32] Soboleva S.E., Ivaschenko V.A., Kostrikina I.A., Nevinsky G.A. (2026). Biochemical Characterization of Ribonuclease Activities from the Sea Cucumber Eupentacta fraudatrix. Front. Biosci..

[33] Sanda A., Kiyomoto M., Iwama M., Ohgi K., Irie M. (2008). Marked changes in the ribonuclease activity of mature and immature gonads of sea urchins *Hemicentrotus pulcherrimus* and *Anthocidaris crassispina*. Biol. Pharm. Bull..

[34] Hyman L.H. (1955). The Invertebrates: Echinodermata: The Coelome Bilateria.

[35] Dolmatov I.Y., Mashanov V.S. (2007). Regeneration in Holothurians.

[36] Dolmatov I.Y. (2009). Regeneration of the digestive system in holothurians. Zh. Obshch. Biol..

[37] Dolmatov I.Y., Kalacheva N.V., Tkacheva E.S., Shulga A.P., Zavalnaya E.G., Shamshurina E.V., Girich A.S., Boyko A.V., Eliseikina M.G. (2021). Expression of *Piwi*, *MMP*, *TIMP*, and *Sox* during Gut Regeneration in Holothurian *Eupentacta fraudatrix* (Holothuroidea, Dendrochirotida). Genes.

[38] Sun L., Sun J., Li X., Zhang L., Yang H., Wang Q. (2017). Understanding regulation of microRNAs on intestine regeneration in the sea cucumber Apostichopus japonicus using high-throughput sequencing. Comp. Biochem. Physiol. Part D Genom. Proteom..

[39] Soboleva S.E., Poletaeva J.E., Dmitrenok P.S., Bulgakov D.V., Ryabchikova E.I., Nevinsky G.A. (2025). Very Stable High-Molecular-Mass Multiprotein Complexes in Different Organs of the Sea Cucumber *Paracaudina chilensis*. Molecules.

[40] Timofeeva A.M., Kostrikina I.A., Dmitrenok P.S., Soboleva S.E., Nevinsky G.A. (2021). Very Stable Two Mega Dalton High-Molecular-Mass Multiprotein Complex from Sea Cucumber *Eupentacta fraudatrix*. Molecules.

[41] Soboleva S.E., Burkova E.E., Dmitrenok P.S., Bulgakov D.V., Menzorova N.I., Buneva V.N., Nevinsky G.A. (2018). Extremely stable high molecular mass soluble multiprotein complex from eggs of sea urchin *Strongylocentrotus intermedius* with phosphatase activity. J. Mol. Recognit..

[42] Soboleva S.E., Dmitrenok P.S., Verkhovod T.D., Buneva V.N., Sedykh S.E., Nevinsky G.A. (2015). Very stable high molecular mass multiprotein complex with DNase and amylase activities in human milk. J. Mol. Recognit..

[43] Burkova E.E., Dmitrenok P.S., Sedykh S.E., Buneva V.N., Soboleva S.E., Nevinsky G.A. (2014). Extremely stable soluble high molecular mass multi-protein complex with DNase activity in human placental tissue. PLoS ONE.

[44] Burkova E.E., Dmitrenok P.S., Bulgakov D.V., Ermakov E.A., Buneva V.N., Soboleva S.E., Nevinsky G.A. (2018). Identification of major proteins of a very stable high molecular mass multi-protein complex of human placental tissue possessing nine different catalytic activities. Biochem. Anal. Biochem..

[45] Alberts B. (1998). The cell as a collection of protein machines: Preparing the next generation of molecular biologists. Cell.

[46] Eubel H., Braun H.P., Millar A.H. (2005). Blue-native PAGE in plants: A tool in analysis of protein-protein interactions. Plant Methods.

[47] Salim D., Gerton J.L. (2019). Ribosomal DNA instability and genome adaptability. Chromosome Res..

[48] Leal G., Comprido D., Duarte C.B., Leal G., Comprido D., Duarte C.B. (2014). BDNF-induced local protein synthesis and synaptic plasticity. Neuropharmacology.

[49] Jiao L., Liu Y., Yu X.Y., Pan X., Zhang Y., Tu J., Song Y.H., Li Y. (2023). Ribosome biogenesis in disease: New players and therapeutic targets. Signal Transduct. Target. Ther..

[50] Gammons M., Bienz M. (2018). Multiprotein complexes governing Wnt signal transduction. Curr. Opin. Cell Biol..

[51] He L., Yang J., Hao Y., Yang X., Shi X., Zhang D., Zhao D., Yan W., Bie X., Chen L. (2023). DDX20: A Multifunctional Complex Protein. Molecules.

[52] Kim J.J., Kingston R.E. (2022). Context-specific Polycomb mechanisms in development. Nat. Rev. Genet..

[53] Arif A., Yao P., Terenzi F., Jia J., Ray P.S., Fox P.L. (2018). The GAIT translational control system. Wiley Interdiscip. Rev. RNA.

[54] Schulte U., den Brave F., Haupt A., Gupta A., Song J., Müller C.S., Engelke J., Mishra S., Mårtensson C., Ellenrieder L. (2023). Mitochondrial complexome reveals quality-control pathways of protein import. Nature.

[55] Swapna L.S., Mahajan S., de Brevern A.G., Srinivasan N. (2012). Comparison of tertiary structures of proteins in protein-protein complexes with unbound forms suggests prevalence of allostery in signalling proteins. BMC Struct. Biol..

[56] Kohrman D.C., Imperiale M.J. (1992). Simian virus 40 large T antigen stably complexes with a 185-kilodalton host protein. J. Virol..

[57] Valach M., Léveillé-Kunst A., Gray M.W., Burger G.J. (2018). Respiratory chain Complex I of unparalleled divergence in diplonemids. Biol. Chem..

[58] Wang F., Wang L., Xu Z., Liang G. (2013). Identification and analysis of multi-protein complexes in placenta. PLoS ONE.

[59] Visscher K.M., Kastritis P.L., Bonvin A.M. (2015). Non-interacting surface solvation and dynamics in protein-protein interactions. Proteins.

[60] Longley M.J., Mosbaugh D.W. (1993). In situ detection of DNA-metabolizing enzyme following polyacrylamide gel electrophoresis. Methods Enzymol..

[61] Vandooren J., Geurts N., Martens E., Van den Steen P.E., Opdenakker G. (2013). Zymography methods for visualizing hydrolytic enzymes. Nat. Methods.

[62] Bencsik P., Bartekova M., Görbe A., Kiss K., Pálóczi J., Radosinska J., Szűcs G., Ferdinandy P. (2017). MMP Activity Detection in Zymograms. Methods Mol. Biol..

[63] Zaksas N.P., Timofeeva A.M., Dmitrenok P.S., Soboleva S.E., Nevinsky G. (2022). Comparison of the Content of Several Elements in Seawater, Sea Cucumber *Eupentacta fraudatrix* and Its High-Molecular-Mass Multiprotein Complex. Molecules.

[64] Suck D. (1994). DNA recognition by DNase I. J. Mol. Recognit..

[65] Znatdinov D.I., Doronina V., Borzova I.V., Mikhailova N.P. (2026). Bioactive components of holothurians: Molecular bases and mechanisms of geroprotective action. Adv. Gerontol..

