# Supplementary material for: Ribonucleases of Very Stable Multi-Protein Complexes from Different Organs of Sea Cucumber Paracaudina chilensis
Source: Int J Mol Sci. 2026 Nov 9;27(21):10051. doi: 10.3390/ijms272110051 (PMC13764322; doi:10.3390/ijms272110051)
Supplement: Supplementary file 1 [file ijms-27-10051-s001.zip › ijms-4602083-supplementary.pdf]

Supplementary data

The data presented in Figures 1 and 2 were obtained from reference [39]: Soboleva, S.E., Poletaeva, J.E., Dmitrenok, P.S., Bulgakov, D.V., Ryabchikova, E.I., Nevinsky, G.A. Very Stable High-Molecular-Mass Multiprotein Complexes in Different Organs of the Sea Cucumber *Paracaudina chilensis*. Molecules. **2025**, 30, 4496. doi: 10.3390/molecules30234496.

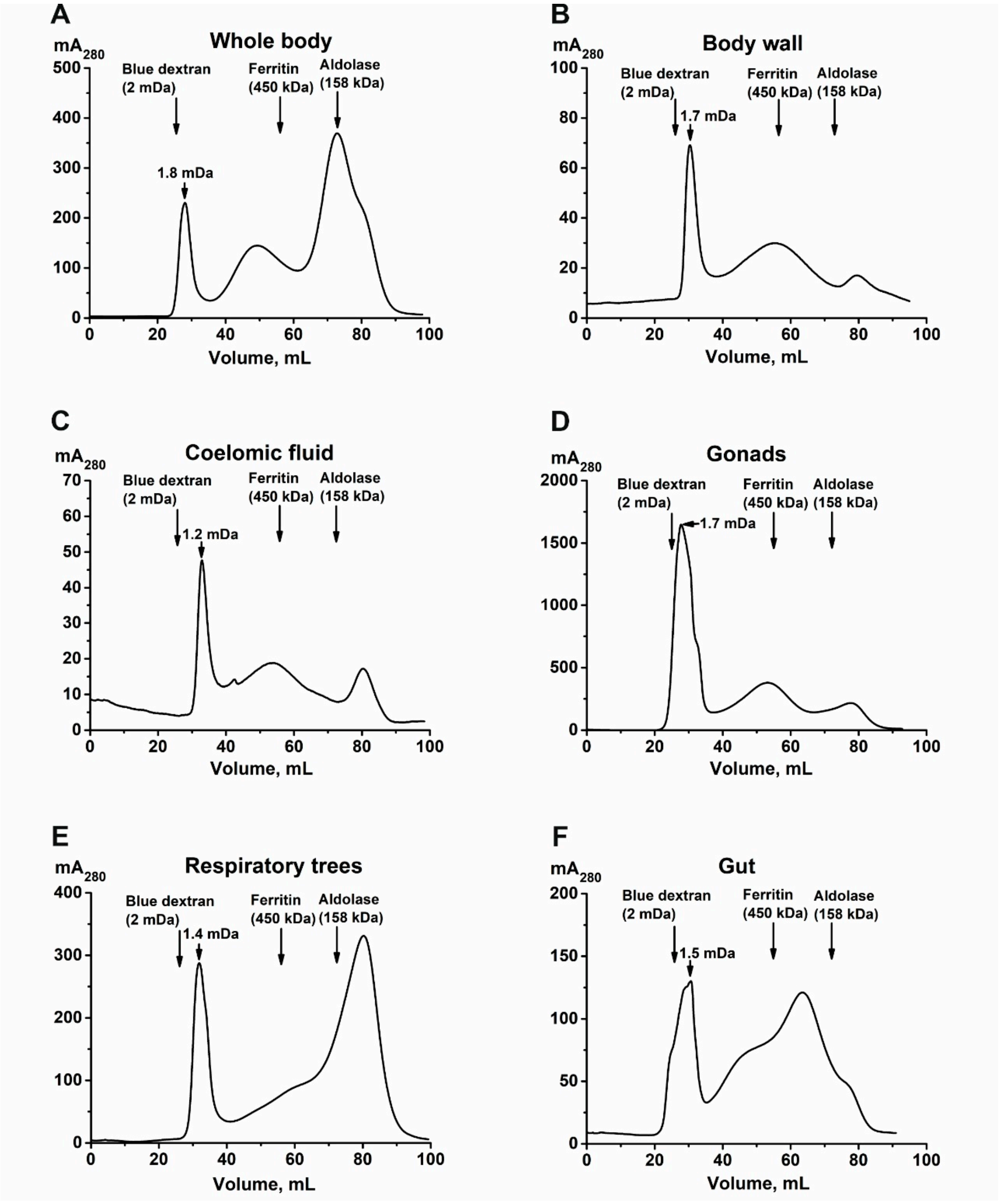

Figure S1. Isolation of protein complexes from the sea cucumber *P. chilensis* by FPLC gel filtration on a Sepharose 4B column using homogenates of intact sea cucumbers (**A**) and their different organs and tissues: body wall (**B**), coelomic fluid (**C**), gonads (**D**), respiratory trees (**E**), and gut (**F**) [39].

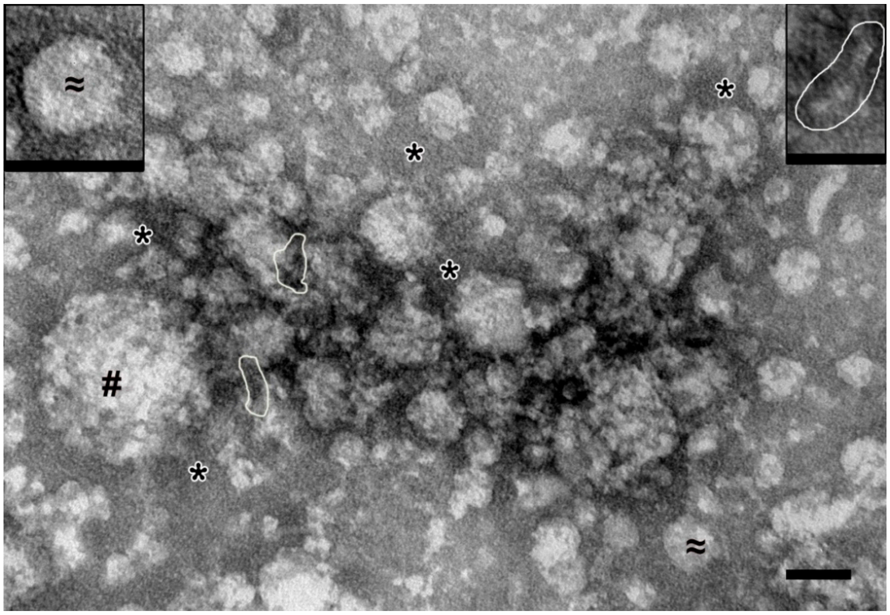

Figure S2. Transmission electron microscopy analysis of complexes. Representative image of complexes obtained by ultracentrifugation from full holothurian body including visceral organs. # - spherical particles of 40-140 nm (protein complexes); ≈ and insert – spherical particles of 30-40 nm (protein complexes); ***** – fine background material; stacks of "sticks" are shown by contour. TEM, negative staining by uranyl acetate. Length of scale bars correspond to 50 nm [39].

Supplementary data to this article:

Ribonucleases of Very Stable Multi-Protein Complexes from Different Organs of Sea Cucumber *Paracaudina chilensis*

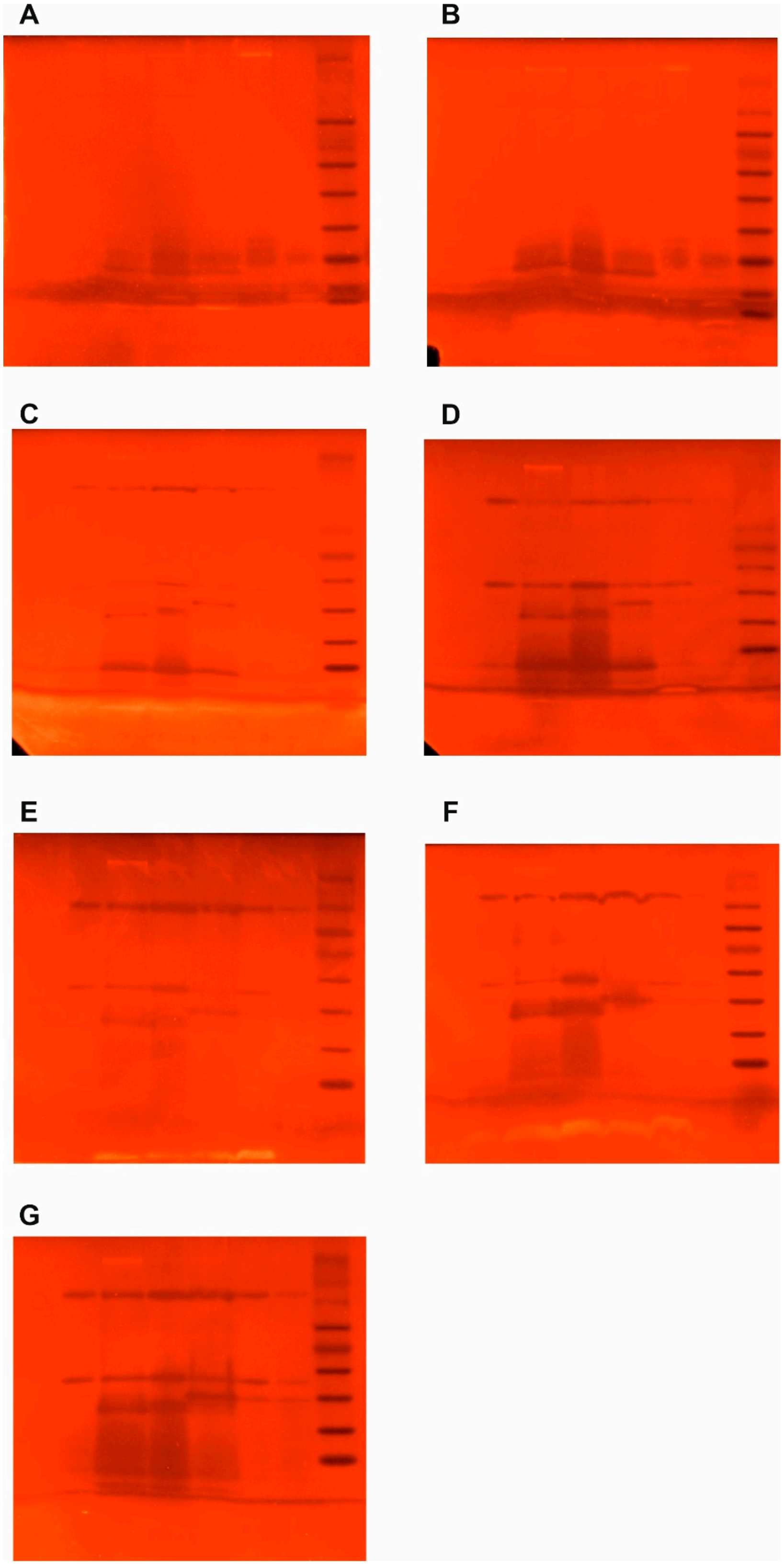
A
B
C
D
E
F
G

Figure S3. Polyacrylamide gels used for RNase activity assay, stained with ethidium bromide. Protein complexes were loaded from left to right in the following order: body wall, coelomic fluid 1, coelomic fluid 2, gut, gonads, and respiratory trees. Protein markers were loaded in the right lane. **(A)** Incubation at pH 3.5. **(B)** Incubation at pH 4.5. **(C)** Incubation at pH 5.5. **(D)** Incubation at pH 6.5. **(E)** Incubation at pH 7.5. **(F)** Incubation at pH 8.5. **(G)** Incubation at pH 9.5.

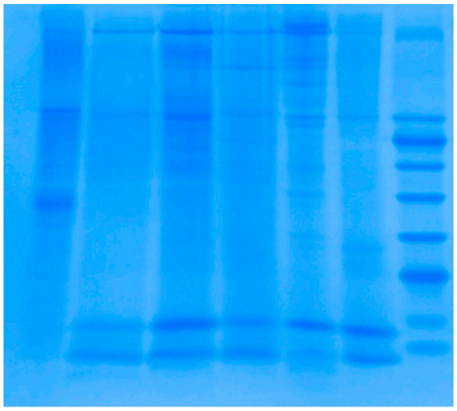

Figure S4. Coomassie-stained polyacrylamide gels after RNase activity assay. Protein complexes were loaded from left to right in the following order: body wall, coelomic fluid 1, coelomic fluid 2, gut, gonads, and respiratory trees. Protein markers were loaded in the right lane. Incubation at pH 5.5.

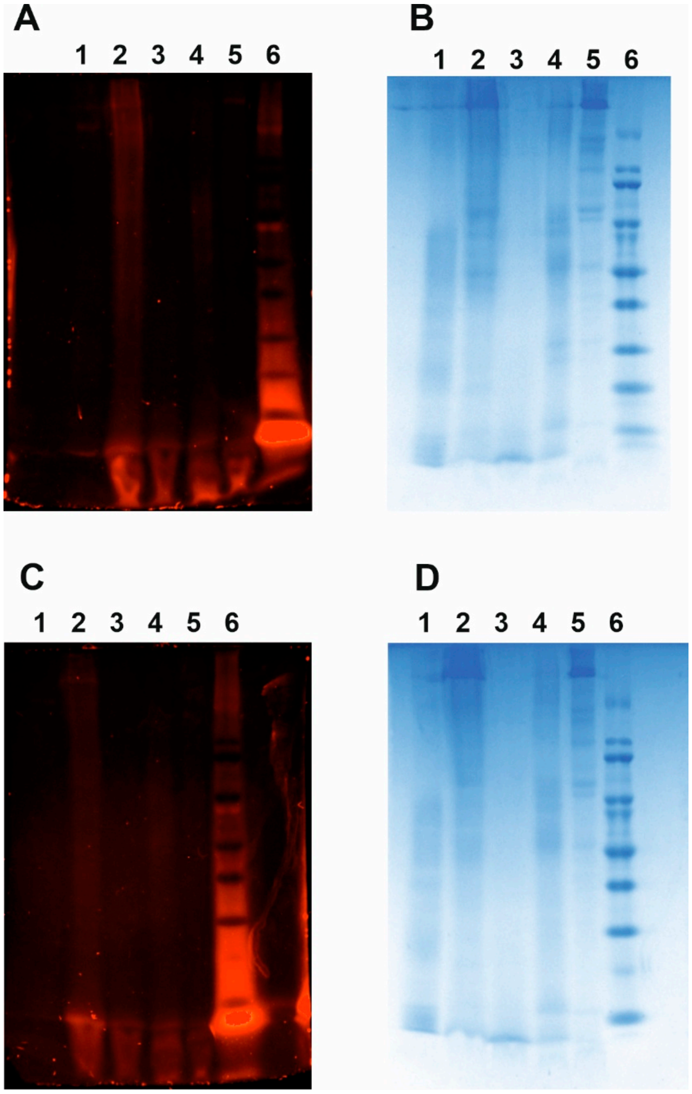

Figure S5. Control experiment in a gel without copolymerized RNA. RNase activity was determined following electrophoretic separation of holothurian complexes. Protein complexes were loaded in the following order: body wall (line 1), coelomic fluid (line 2), gut (line 3), respiratory trees (line 4), and gonads (line 5). Incubation at pH 5.5 (**A** and **B**) and pH 8.5 (**C** and **D**). Gels were stained with ethidium bromide (**A** and **C**) and Coomassie Blue (**B** and **D**).

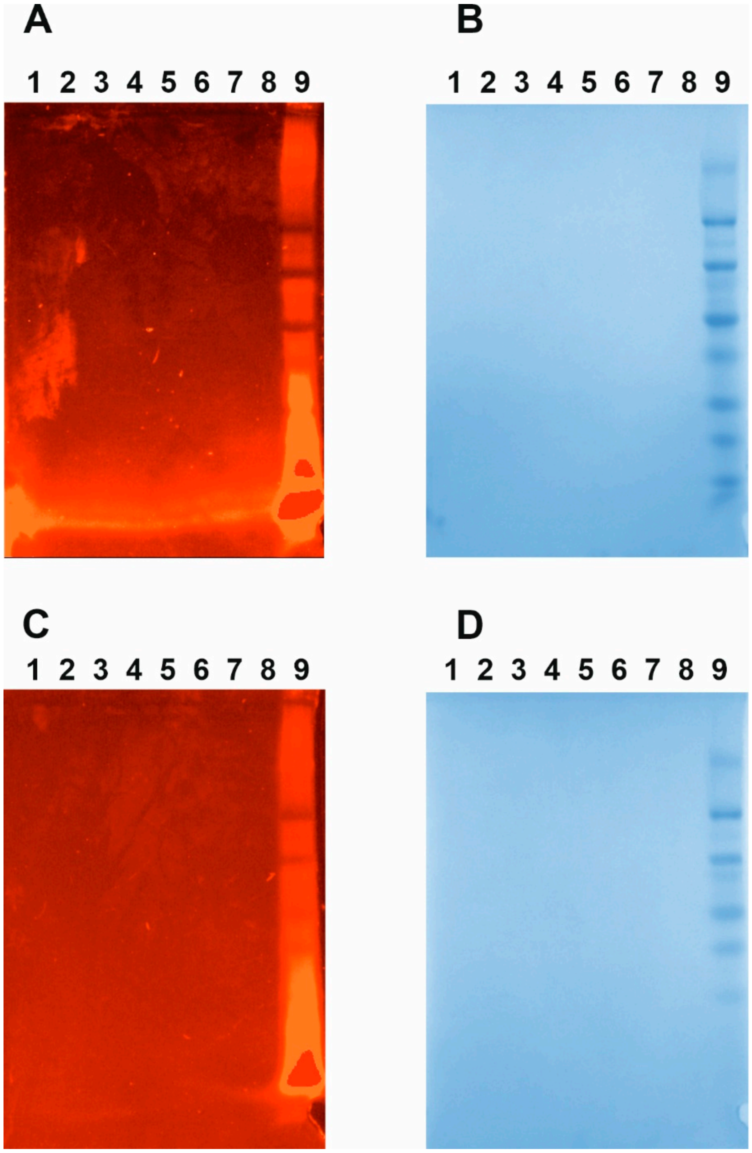

Figure S6. Control experiment in a polyacrylamide gel containing copolymerized RNA, without loading protein complexes. Samples were loaded in the following order: lane 1, 20 mM MES-NaOH buffer, pH 5.5 (for panels **A** and **B**) or 20 mM Tris-HCl buffer, pH 8.5 (for panels **C** and **D**); lane 2, 20 mM $MgCl_2$; lane 3, 20 mM $CaCl_2$; lane 4, 20 mM $MnCl_2$; lane 5, 20 mM $CoCl_2$; lane 6, 20 mM $NiCl_2$; lane 7, 20 mM $ZnCl_2$; lane 8, 20 mM EDTA; lane 9, molecular weight markers. After electrophoretic separation, RNase activity was determined. Incubation was performed at pH 5.5 (panels **A** and **B**) and at pH 8.5 (panels **C** and **D**). The gels were stained with ethidium bromide (panels **A** and **C**) and Coomassie Blue (panels **B** and **D**).

Supplementary Methods S1.

The data from reference : Soboleva, S.E., Poletaeva, J.E., Dmitrenok, P.S., Bulgakov, D.V., Ryabchikova, E.I., Nevinsky, G.A. Very Stable High-Molecular-Mass Multiprotein Complexes in Different Organs of the Sea Cucumber *Paracaudina chilensis*. Molecules. **2025**, 30, 4496. doi: 10.3390/molecules30234496.

Complex separation and purification

For removing possible stable protein complexes, the extract of intact sea cucumber *P. chilensis* was subjected to gel filtration on Sepharose 4B, efficiently separating various proteins with molecular weights (MWs) of 60–20000 kDa. The concentrated protein preparations (1 mL) were applied on a column with Sepharose 4B (90 mL) equilibrated in TBS buffer (20 mM Tris HCl pH 7.5, 0.5 M NaCl, and all antibiotics) using chromatograph GE Akta Purifier (Chicago, IL, USA) and fractions (4.0 mL) eluted by the same buffer were collected. The complex and other proteins were monitored by absorbance at 280 nm (A_{280}). Isolation of stable complexes from various organs (body wall, gut, respiratory trees, and gonads) was carried out using the same conditions described above for the whole organism. For removing NaCl all fractions were dialyzed against 10 mM Tris-HCl, pH 7.5 for 16 hours at 4° C and were concentrated on a rotary evaporator (Refrigerated Centri Vap Concentrator, Labconco, USA). For search of the multi-protein complexes, all holothurian preparations corresponding to the first peak after gel filtration were used. Ultracentrifugation at 100,000g for 2 hours was used to purify the preparations from various vesicles and cell membrane fragments (Optima XE-90 Ultracentrifuge; Beckman Coulter, California, USA) Then supernatants used for following different types of analysis. All experiments were performed under sterile conditions.

Each of the samples was applied for 1 min on a copper grid covered using formvar film stabilized with carbon. Then grids were placed for 7–10 sec on a drop of 0.5 % water solution of uranyl acetate. Then liquids were removed using filter paper. All grids were analyzed in a transmission electron microscope Jem1400 (Jeol, Tokyo, Japan), and the images obtained were collected using a Veleta digital camera (EM SIS, Muenster, Germany). The measurements were made using the ITEM software version 5.2 (EM SIS, Muenster, Germany).

Table S1 from reference : Soboleva, S.E., Poletaeva, J.E., Dmitrenok, P.S., Bulgakov, D.V., Ryabchikova, E.I., Nevinsky, G.A. Very Stable High-Molecular-Mass Multiprotein Complexes in Different Organs of the Sea Cucumber *Paracaudina chilensis*. Molecules. **2025**, 30, 4496. doi: 10.3390/molecules30234496.

Table S1. Molecular masses (m/z) of 10–20 kDa proteins determined by MALDI-mass spectromenty in stable multiprotein complexes from different organs of the sea cucumber *P. chilensis* (kDa).

	Whole organism	Body wall (11 proteins)	Respiratory trees (12 proteins)	Gut (12 proteins)	Celomic fluid (15 proteins)	Gonads (7 proteins)
1	18.566 ± 2.4	+	+	+	+	+
2	15.066 ± 2.4	+	+	+	+	+
3	14.920 ± 2.1	–	–	–	+	–
4	14.794 ± 2.1	–		–	+	–

5	14.694.2 ± 2.1	+	+	–	+	+
6	13.559.0 ± 2.4	–	–	–	+	–
7	12.180.7 ± 2.8	–	–	+	–	–
8	11.743.6 ± 2.0	–	–	+	–	–
9	11.737.4 ± 1.7	–	+	–	–	–
10	11.733.5 ± 1.5	+	-	+	+	+
11	11.729.8 ± 2.5	–	+	–	–	–
12	11.725.7 ± 3.1	+	+	–	–	–
13	11.721.2 ± 2.2	+	–	+	–	–
14	11.716.1 ± 1.6	–	–	+	+	+
15	11.713.8 ± 2.3	+	+	–	–	–
16	11.660.3 ± 2.8	–	–	+	–	–
17	11.546.0 ± 1.9	–	+	+	–	–
18	11.526.0 ± 2.1	–	+	+	–	–
19	11.523.6 ± 1.8	+	–	+	+	–
20	11.393.6 ± 2.1	–	–	–	+	–
21	11.373.6 ± 1.6	–	+	–	+	+
22	11.242.7 ± 1.9				+	
23	11.178.3 ± 2.2	–	+	–	–	–
24	11.054 ± 1.9				+	
25	10.883.5 ± 1.2	–	+	+	+	+
26	10.859.6 ± 2.9	+	–	–	–	–
27	10.767.8 ± 2.4	+	–	–	+	–
28	10.456 ± 1.9	+	–	–	–	–

Table S2 from reference : Soboleva, S.E., Poletaeva, J.E., Dmitrenok, P.S., Bulgakov, D.V., Ryabchikova, E.I., Nevinsky, G.A. Very Stable High-Molecular-Mass Multiprotein Complexes in Different Organs of the Sea Cucumber *Paracaudina chilensis*. Molecules. **2025**, 30, 4496. doi: 10.3390/molecules30234496.

Supplementary Table S2. Comparison of peptides of multuiprotein complexes from different organs of the see cucumber *P. chilensis*

Molecular weights of peptides in Daltons (**the assigned number for the peptide in particular organ*****) ****						
**Respiratory trees (104 peptides)**	***Body wall*** **(64 peptides)**	***Gut*** **(58 peptides)**	**Coelomic fluid (76 peptides)**	**Gonads (55 peptides)**	**№**	**All peptides In all organs**
		8900.2 *(1)*			**1**	**8900.2**
8893.2 (1)					**2**	***8893.2***
		8399.4 *(2)*			**3**	**8399.4**
8224.1 (2)					**4**	***8224.1***
8220.4 *(3)*				8220.2 *(1)*	**5**	**8220.4**
8217.2 *(4)*			8217.2 *(1)*		**6**	**8217.2**
				8213.7 *(2)*	**7**	**8213.7**
		8197.8 *(3)*			**8**	**8197.8**
				8148.4 *(3)*	**9**	**8148.4**
	7905.8 *(1)*				**10**	**7905.8**
		7664.4 *(4)*	7664.4 *(2)*		**11**	**7664.4**
		7660.9 *(5)*			**12**	**7660.9**
		7637.9 *(6)*			**13**	**7637.9**
7620.5 *(5)*	7620.5 *(2)*	7620.5 *(7)*			**14**	**7620.5**
			7624.1 *(3)*		**15**	**7624.1**
			7616.5 *(4)*		**16**	**7616.5**
7614.7 *(6)*				7614.7 *(4)*	**17**	**7614.7**
7612.4 *(7)*		7612.4 *(8)*	7612.4 *(5)*		**18**	**7612.4**
7610.4 *(8)*			7610.4 *(6)*	7610.4 *(5)*	**19**	**7610.4**
		7606.4 *(9)*			**20**	**7610.4**
			7529.8 *(7)*		**21**	**7529.8**
	7453.3 *(3)*				**22**	**7453.3**
			7035.1 *(8)*		**23**	**7035.1**
7432.3 *(9)*					**24**	**7432.3**
7420.8 *(10)*		7020.5 *(10)*			**25**	**7420.8**

7413.9 *(11)*					**26**	**7413.9**
7406.0 *(12)*					**27**	**7406.0**
7387.5 *(13)*					**28**	**7387.5**
7365.2 *(14)*					**29**	**7365.2**
7 62.9 *(15)*				7362.9 *(6)*	**30**	**7 62.9**
	7358.3. *(4)*		7358.3 *(9)*		**31**	**7358.3**
7357.3 *(16)*		7357.3 *(11)*			**32**	**7357.3**
			7355.1 *(10)*		**33**	**7355.1**
7353.7 *(17)*		7353.7 *(12)*		7353.7 *(7)*	**34**	**7353.7**
7351.0 *(18)*	7351.0 *(5)*			7351.0 *(8)*	**35**	**7351.0**
7347.7 *(19)*		7347.7 *(13)*	7347.7 *(11)*	7348.7 *(9)*	**36**	**7347.7**
7345.6 *(20)*	7345.6 *(6)*	7345.6 *(14)*	7345.6 *(12)*		**37**	**7345.6**
		7342.3 *(15)*			**38**	**7342.3**
7337.8 *(21)*					**39**	**7337.8**
				7336.1 *(10)*	**40**	**7336.1**
		7332.1 *(16)*	7332.1 *(13)*		**41**	**7332.1**
		7326.5 *(17)*			**42**	**7326.5**
7108.9 *(22)*					**43**	**7108.9**
7099.5 *(23)*					**44**	**7099.5**
7091.3 *(24)*					**45**	**7091.3**
				7051.1 *(11)*	**46**	**7051.1**
		7043.9 *(18)*			**47**	**7043.9**
7035.7 *(25)*					**48**	**7035.7**
				7032.3 *(12)*	**49**	**7032.3**
7030.3 *(26)*					**50**	**7030.3**
			7025.5 *(14)*		**31**	**7025.5**
			703.8 *(15)*		**52**	**7023.8**
	7021.5 *(7)*	7021.5 *(19)*	7021.5 *(16)*		**53**	**7021.5**
7015.5 *(27)*		7015.5 *(20)*	7016.5 *(17)*	7015.5 *(13)*	**54**	**7015.5**
			7012.7		**55**	**7012.7**

			(18)			
	7010.5 *(8)*				**56**	**7010.5**
7006.7 *(28)*	7006.7 *(9)*	7006.7 *(21)*			**57**	**7006.7**
		7003.6 *(22)*			**58**	**7003.6**
			7002.8 *(19)*		**59**	**7002.8**
	7001.3 *(10)*				**60**	**7001.3**
6998.8 *(29)*					**61**	**6998.8**
	6995.3 *(11)*		6995.3 *(20)*	6995.3 *(14)*	**62**	**6995.3**
6991.7 *(30)*		6991.7 *(23)*			**63**	**6991.7**
			6989.2 *(21)*		**64**	**6989.2**
	6988.5 *(12)*		6988.5 *(22)*		**65**	**6988.5**
6977.7 *(31)*			6977.7 *(23)*		**66**	**6977.7**
			6976.5 *(24)*	6976.5 *(15)*	**67**	**6976.5**
6974.7 *(32)*	6974.7 *(13)*	6974.7 *(24)*			**68**	**6974.7**
6970.5 *(33)*		6970.5 *(25)*		6970.5 *(16)*	**69**	**6970.5**
6967.2 *(34)*					**70**	**6967.2**
				6965.6 *(17)*	**71**	**6965.6**
	6957.2 *(14)*				**72**	**6957.2**
6955.6 *(35)*			6955.6 *(25)*		**73**	**6955.6**
**6953.4 *(36)***	**6953.4 *(15)***	**6953.4 *(26)***	**6953.9 *(26)***	**6953.4 *(18)***	**74**	**6953.4**
6952.5 *(37)*			6952.5 *(27)*		**75**	**6952.5**
		6950.4 *(27)*			**76**	**6950.4**
				6946.2 *(19)*	**77**	**6946.2**
	6933.3 *(16)*				**78**	**6933.3**
			6926.3		**79**	**6926.3**

			(28)			
		6911.1 *(28)*			**80**	**6911.1**
			6828.0 *(29)*		**81**	**6828.0**
6824.6 *(38)*					**82**	**6824.6**
		6920.8 *(29)*			**83**	**6920.8**
		6826.4 *(30)*			**84**	**6826.4**
	6822.5 *(17)*				**85**	**6822.5**
6815.5 *(39)*					**86**	**6815.5**
	6602.8 *(18)*		6602.8 *(30)*		**87**	**6602.8**
			6521.9 *(31)*		**88**	**6521.9**
	6468.2 *(19)*				**89**	**6468.2**
6411.5 *(40)*					**90**	**6411.5**
6354.3 *(41)*					**91**	**6354.3**
		6289.2 *(31)*			**92**	**6289.2**
			6066.3 *(32)*		**93**	**6066.3**
6045.3 *(42)*			6045.3 *(33)*		**94**	**6045.3**
			6036.5 *(34)*		**95**	**6036.5**
			6028.4 *(35)*		**96**	**6028.4**
6025.0 *(43)*	6025.9 *(20)*		6025.9 *(36)*	6025.9 *(20)*	**97**	**6025.9**
				6024.3 *(21)*	**98**	**6024.3**
6023.3 *(44)*	6023.3 *(21)*		6023.3 *(37)*	6023.3 *(22)*	**99**	**6023.3 6**
6021.7 *(45)*		6021.7 *(32)*			**100**	**6021.7**
6019.9 *(46)*	6019.9 *(22)*				**101**	**6019.9**
		6018.2 *(33)*			**102**	**6019.9**
			6011.1		**103**	**6011.1**

			(38)			
				6001.6 *(23)*	**104**	**6001.6**
5853.5 *(47)*				5853.5 *(24)*	**105**	**5853.5**
	5805.6 *(23)*				**106**	**5805.6**
5786.3 *(48)*					**107**	**5786.3**
**5757.5 *(49)***	**5757.5 *(24)***	**5757.5 *(34)***	**5757.5 *(39)***	**5757.5 *(25)***	**108**	**5757.5**
	5754.5 *(25)*		5754.5 *(40)*		**109**	**5754.5**
	5707.3 *(26)*				**110**	**5707.3**
	5686.7 *(27)*	5686.7 *(35)*			**111**	**5686.7**
			5683.6 *(41)*		**112**	**5683.6**
			5681.5 *(42)*	5681.5 *(26)*	**113**	**5681.5**
			5680.0 *(43)*		**114**	**5680.0**
5589.4 *(51)*			5589.4 *(44)*	5589.4 *(27)*	**115**	**5589.4**
	5587.1 *(29)*		5587.1 *(45)*		**116**	**5587.1**
5582.2 *(52)*				5582.2 *(28)*	**117**	**5582.2**
				5676.6 *(29)*	**118**	**5676.6**
5526.4 *(53)*	5526.4 *(30)*			5526.4 *(30)*	**119**	**5526.4**
5523.7 *(54)*	5523.7 *(31)*		5523.7 *(46)*		**120**	**5523.7**
			5522.5 *(47)*		**121**	**5522.5**
5467.0 *(55)*					**122**	**5467.0**
5460.8 *(56)*					**123**	**5460.8**
5446.5 *(57)*					**124**	**5446.5**
5441.3 *(58)*					**125**	**5441.3**
			5435.6 *(48)*		**126**	**5435.6**
			5434.0 *(49)*	5434.0 *(31)*	**127**	**5434.0**
			5427.1 *(50)*		**128**	**5427.1**
5425.3 *(59)*					**129**	**5425.3**

	5423.3 *(32)*				**130**	**5423.3**
5436.6 *(60)*					**131**	**5436.6**
5418.7 *(61)*					**132**	**5418.7**
5403.7 *(62)*					**133**	**5403.7**
5400.3 *(63)*					**134**	**5400.3**
5394.0 *(64)*					**135**	**5394.0**
			5379.7 *(51)*		**136**	**5379.7**
5281.7 *(65)*	5379.7 *(33)*	5379.7 *(36)*			**137**	**5379.7**
5376.3 (66)	5376.3 *(34)*	5276.3 *(37)*			**138**	**5276.3**
		5375.1 *(38)*	5375.1 *(52)*	5375.1 *(32)*	**139**	**5375.1**
5373.6 *(67)*	5373.6 *(35)*			5373.6 *(33)*	**140**	**5373.6**
5306.0 *(68)*					**141**	**5306.0**
5302.3 *(69)*					**142**	**5302.3**
5298.9 *(70)*					**143**	**5298.9**
5294.0 *(71)*					**144**	**5294.0**
5284.3 *(72)*					**145**	**5284.3**
5278.7 *(73)*		5278.7 *(39)*			**146**	**5278.7**
5277.1 *(74)*			5277.1 *(53)*	5277.1 *(34)*	**147**	**5277.1**
	5268.5 *(36)*				**148**	**5268.5**
	5266.5 *(37)*				**149**	**5266.5**
5254.6 *(75)*					**150**	**5254.6**
5247.7 *(76)*					**151**	**5247.7**
5229.8 *(77)*	5229.8 *(38)*		5229.8 *(54)*	5229.8 *(35)*	**152**	**5229.8**
**5227.5 *(78)***	**5227.5 *(39)***	**5227.5 *(40)***	**5227.5 *(55)***	**5227.5 *(36)***	**153**	**5227.5**
5230.1 *(79)*					**154**	**5230.1**
5225.3 *(80)*		5225.3 *(41)*			**155**	**5225.3**
	5220.9 *(40)*				**156**	**5220.9**
			5215.7 *(56)*		**157**	

	5192.7 *(41)*				**158**	**5192.7**
	5183.7 *(42)*				**159**	**5183.7**
	5171.7 *(43)*				**160**	**5171.7**
	5164.9 *(44)*				**161**	**5164.9**
5150.0 *(81)*					**162**	**5150.0**
			5147.9 *(57)*	5147.9 *(37)*	**163**	**5147.9**
		5145.6 *(42)*			**164**	**5145.6**
				5135.7 *(38)*	**165**	**5135.7**
				5130.1 *(39)*	**166**	**5130.1**
	5098.2 *(45)*				**167**	**5098.2**
	5093.1 *(46)*				**168**	**5093.1**
		5045.2 *(43)*			**169**	**5045.2**
	4967.6 *(47)*				**170**	**4967.6**
		4925.7 *(44)*			**171**	**4925.7**
	4903.1 *(48)*				**172**	**4903.1**
	4833.1 *(49)*				**173**	**4833.1**
					**174**	
4815.2 *(82)*					**175**	**4815.2**
			4810.2 *(58)*		**176**	**4810.2**
	4669.4 *(50)*				**177**	**4669.4**
4573.7 *(83)*					**178**	**4573.7**
				4388.8 *(40)*	**179**	**4388.8**
	4368.7 *(51)*				**180**	**4368.7**
		4310.5 *(45)*			**181**	**4310.5**
		4293.0 *(46)*			**182**	**4293.0**
	4214.7 *(52)*				**183**	**4214.7**

	4206.2 *(53)*				**184**	**4206.2**
4147.0 *(84)*					**185**	**4147.0**
4142.8 *(85)*					**186**	**4142.8**
4120.8 *(86)*	4120.8 *(54)*		4120.8 *(59)*	4120.8 *(41)*	**187**	**4120.8**
4117.0 *(87)*					**188**	**4117.0**
	4055.5 *(55)*				**189**	**4055.5**
				4052.3 *(42)*	**190**	**4052.3**
4039.4 *(88)*			4039.4 *(60)*	4039.4 *(43)*	**191**	**4039.4**
				4035.3 *(44)*	**192**	**4035.3**
4033.6 *(89)*					**193**	**4033.6**
				4030.9 *(45)*	**194**	**4030.9**
4016.6 *(90)*					**195**	**4016.6**
				4014.7 *(46)*	**196**	**4014.7**
4012.4 *(91)*			4012.4 *(61)*		**197**	**4012.4**
				4010.7 *(47)*	**198**	**4010.7**
3997.3 *(92)*					**199**	**3997.3**
3995.3 *(93)*			3995.3 *(62)*		**200**	**3995.3**
			3994.6 *(63)*		**201**	**3994.6**
	3991.0 *(56)*				**202**	**3991.0**
	3977.9 *(57)*				**203**	**3977.9**
3973.4 *(94)*			3973.4 (64)		**204**	**3973.4**
**3971.8 *(95)***	**3971.8 *(58)***	**3971.8 *(47)***	**3971.8 *(65)***	**3971.8 *(48)***	**205**	**3971.8**
3962.8 *(96)*	3962.8 *(59)*				**206**	**3962.8**
	3960.1 *(60)*				**207**	**3960.1**
3878.3 *(97)*					**208**	**3878.3**
3874.4 *(98)*					**209**	**3874.4**
3871.9 *(99)*					**210**	**3871.9**
		3869.1 *(48)*			**211**	**3869.1**
			3841.4 *(66)*		**212**	**3841.4**

			3836.9 *(67)*		**213**	**3836.9**
		3829.4 *(49)*			**214**	**3713.1**
		3827.5 *(50)*			**215**	**3713.1**
				3825.7 *(49)*	**216**	**3825.7**
			3819.9 *(68)*		**217**	**3819.9**
			3817.0 *(69)*		**218**	**3817.0**
**3815.3** ***(100)***	**3815.2** ***(61)***	**3814.5** ***(51)***	**3814.5** ***(70)***	**3814.5** ***(50)***	**219**	**3814.5**
			3812.4 *(71)*	3812.4 *(51)*	**220**	**3812.4**
		3798.6 *(52)*			**221**	**3798.6**
			3718.9 *(72)*		**222**	**3718.9**
	3713.1 *(62)*				**223**	**3713.1**
3711.6 *(101)*					**224**	**3711.6**
		3704.5 *(53)*			**225**	**3704.5**
		3692.2 *(54)*			**226**	**3692.2**
	3625.5 *(63)*				**227**	**3625.5**
	3524.4 *(64)*				**228**	**3524.4**
		3517.6 *(55)*			**229**	**3517.6**
3509.7 *(102)*					**230**	**3509.7**
3473.2 *(103)*		3473.2 *(56)*			**231**	**3473.2**
			3465.9 *(73)*	3465.9 *(52)*	**232**	**3465.9**
			3463.1 *(74)*	3463.1 *(53)*	**233**	**3463.1**
3462.6 *(104)*		3462.7 *(57)*	3462.7 *(75)*	3462.7 *(54)*	**234**	**3462.7**
			3455.7 *(76)*		**235**	**3455.7**
				3283.2 *(55)*	**236**	**3283.2**
		2996.5			**237**	**2996.5**

		(58)				

*Table S2 contains only those very close MWs, which corresponded to closely spaced peaks in the same spectrum. Five major peptides (numbers 74, 108, 153, 205, and 219) are highlighted in bold.
